# NK cells require immune checkpoint receptor LILRB4/gp49B to control neurotropic Zika virus infections in mice

**DOI:** 10.1172/jci.insight.151420

**Published:** 2022-02-08

**Authors:** Ha-Na Lee, Mohanraj Manangeeswaran, Aaron P. Lewkowicz, Kaliroi Engel, Monica Chowdhury, Mamatha Garige, Michael A. Eckhaus, Carole Sourbier, Derek D.C. Ireland, Daniela Verthelyi

**Affiliations:** 1 Laboratory of Immunology, Center of Excellence in Infectious Disease and Inflammation, Office of Biotechnology Products, and; 2 Laboratory of Molecular Oncology, Division of Biotechnology Review and Research-I, Office of Biotechnology Products, Office of Pharmaceutical Quality, Center for Drug Evaluation and Research (CDER), US Food and Drug Administration (FDA), Silver Spring, Maryland, USA.; 3 Division of Veterinary Resources, Office of Research Services, National Institutes of Health (NIH), Bethesda, Maryland, USA.

**Keywords:** Immunology, Infectious disease, NK cells

## Abstract

Immune cells express an array of inhibitory checkpoint receptors that are upregulated upon activation and limit tissue damage associated with excessive response to pathogens or allergens. Mouse leukocyte immunoglobulin like receptor B4 (LILRB4), also known as glycoprotein 49B (gp49B), is an inhibitory checkpoint receptor constitutively expressed in myeloid cells and upregulated in B cells, T cells, and NK cells upon activation. Here, we report that expression of LILRB4, which binds Zika virus (ZIKV), was increased in microglia and myeloid cells infiltrating the brains of neonatal mice with ZIKV-associated meningoencephalitis. Importantly, while C57BL/6 mice developed transient neurological symptoms but survived infection, mice lacking LILRB4/gp49B (LILRB4 KO) exhibited more severe signs of neurological disease and succumbed to disease. Their brains showed increased cellular infiltration but reduced control of viral burden. The reduced viral clearance was associated with altered NK cell function in the absence of LILRB4/gp49B. In naive animals, this manifested as reduced granzyme B responses to stimulation, but in ZIKV-infected animals, NK cells showed phenotypic changes that suggested altered maturation, diminished glucose consumption, reduced IFN-γ and granzyme B production, and impaired cytotoxicity. Together, our data reveal LILRB4/gp49B as an important regulator of NK cell function during viral infections.

## Graphical abstract

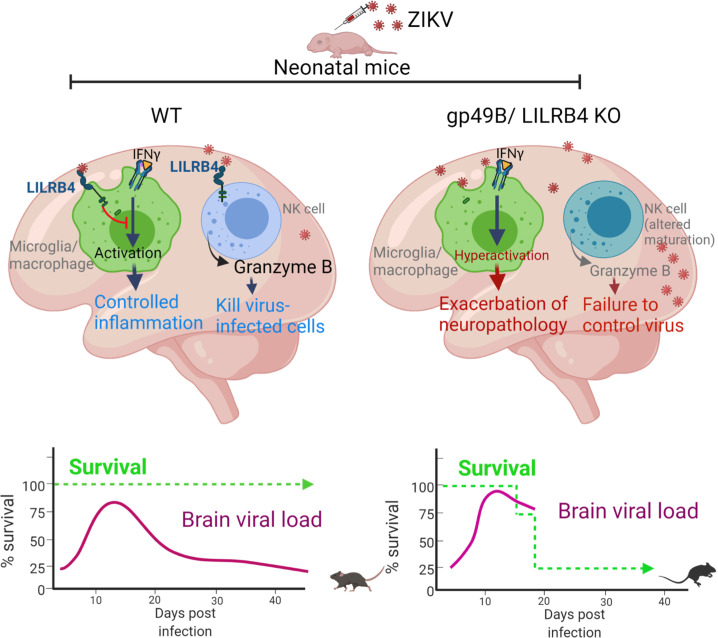

## Introduction

Immune checkpoint receptors are expressed on immune cells upon activation and play a critical role in maintaining self-tolerance and controlling the magnitude and length of immune responses, thereby protecting tissues from immune-driven damage (1, 2). Several studies have shown that some pathogens can promote inhibitory interactions through immune checkpoint proteins to downregulate lymphocyte activity and escape immune control (3, 4). This suggests that their therapeutic blockade could be extended from the treatment of cancer to infectious diseases, but experience is limited (5, 6).

Antibodies blocking leukocyte immunoglobulin like receptor B4 (LILRB4) are being tested as therapeutics for cancer (7, 8). Murine LILRB4 (also known as glycoprotein 49B, gp49B), is a type I transmembrane glycoprotein bearing 2 tyrosine-based inhibitory motifs (ITIMs) in its intracellular tail. This is different from human LILRB4, which has 3 ITIMs, but both murine and human LILRB4 function as inhibitory checkpoint receptors (9). In mice, LILRB4 is constitutively expressed on myeloid cells, including macrophages, mast cells, and neutrophils, and induced upon activation on subsets of B cells, NK cells, and T cells (9), and its sole known ligand in mice is integrin α_v_β_3_ (10). It negatively regulates immune responses by preventing excessive cell activation (10, 11) and reducing cytokine (12–14) and antibody production (15). Consistent with this, mice lacking LILRB4 have increased vascular injury induced by lipopolysaccharide (LPS) (16), exaggerated stem cell factor–induced mast cell activation (17) and inflammatory responses to type II collagen monoclonal antibodies and LPS (13), as well as more severe allergic inflammatory responses in a ragweed model (18) and accelerated development of atherosclerotic lesions (19), reaffirming its regulatory function. Interestingly, a few reports document increased expression of LILRB4 during acute infection with lymphocytic choriomeningitis virus (LCMV), influenza, and severe acute respiratory syndrome coronavirus 2 (SARS-CoV-2) (3, 20, 21), and its expression is associated with robust antibody responses in patients with influenza (20) as well as development of effector CD8^+^ T cells in mice (14). In addition, a report suggests increased LILRB4 in activated NK cells during CMV infection and enhanced IFN-γ production by T cells and NK cells upon infection with vaccinia (12, 14). However, the role of LILRB4 in regulating the response to viral infection is mostly unknown.

Control of immune-mediated damage is critical in central nervous system (CNS) infections as neurons are terminally differentiated, and cell death can lead to neurological deficits. To better understand the role of LILRB4 in regulating the acute response to viral meningoencephalitis, we examined its role in a model of Zika virus (ZIKV). In a previous study, we established that infection of neonatal C57BL/6 mice with ZIKV results in a transient meningoencephalitis characterized by unsteady gait, kinetic tremors, severe ataxia, and seizures that peak at 12–15 days postinfection (dpi) and subside 10 days later (22). Using the same model, here we show increased expression of LILRB4 in the brains of ZIKV-infected mice and investigate the role of LILRB4 in controlling tissue damage and survival.

## Results

### LILRB4 expression is upregulated on microglia and infiltrating myeloid cells during ZIKV infection.

**Figure 1:**
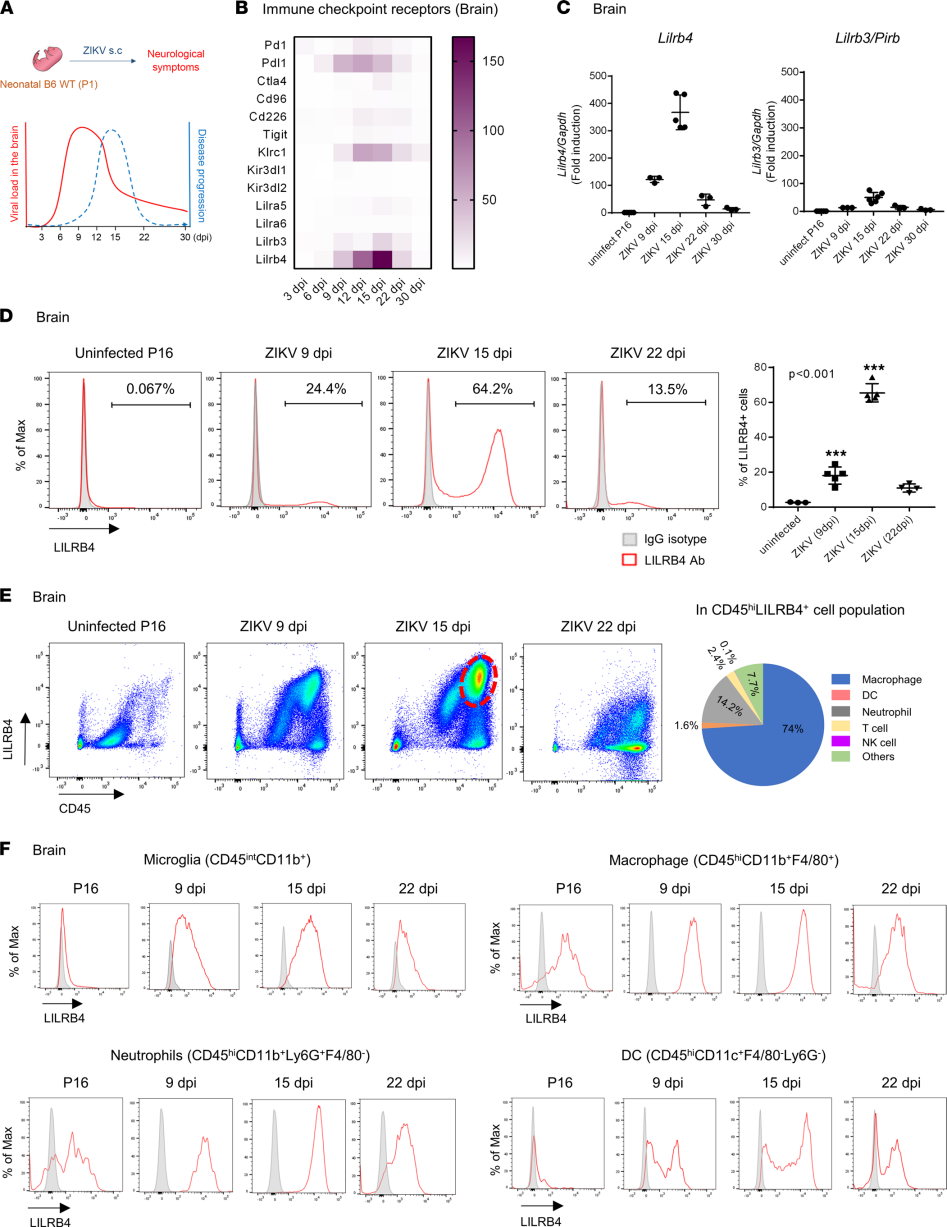
LILRB4 expression is upregulated by microglia and infiltrated myeloid cells into brains during ZIKV infection. (**A**) Diagram depicting the disease progression of ZIKV infection. (**B**) Heatmap for the fold changes in RNA expression of immune checkpoint receptors in the brains of ZIKV-infected mice at the indicated time points (*n* = 3–8, each time point). RNA expression was assessed by NanoString analysis using the nCounter mouse immunology panel. (**C**) Relative mRNA levels of *Lilrb4* and *Lilrb3* in the mouse brain during ZIKV infection compared with uninfected mouse brain. (**D**–**F**) Flow cytometry analysis of LILRB4 expression on cells isolated from the brains of ZIKV-infected mice at the indicated time points. The graph shows the percentage of LILRB4-expressing cells in total live cells (**D**). Live cells were separated based on CD45 and LILRB4 expression. The pie chart shows the percentage of macrophages (CD45^hi^CD11b^+^F4/80^+^), neutrophils (CD45^hi^CD11b^+^Ly6G^+^F4/80^–^), DCs (CD45^hi^CD11c^+^F4/80^–^Ly6G^–^), T cells (CD45^hi^CD3^+^NK1.1^–^), and NK cells (CD45^hi^ NK1.1^+^CD3^–^) within CD45^hi^LILRB4^+^ cell lation (indicated by the dashed circle) at 15 dpi (**E**). The histogram shows LILRB4 expression on microglia (CD45^lo^CD11b^+^), macrophages (CD45^hi^CD11b^+^F4/80^+^), neutrophils (CD45^hi^CD11b^+^Ly6G^+^F4/80^–^), and DCs (CD45^hi^CD11c^+^F4/80^–^Ly6G^–^). Gray histograms indicate staining with IgG isotype control antibodies (**F**). Data are representative of 2 independent experiments (*n* = 3–5, each time point) (**E** and **F**). Data were analyzed using 1-way ANOVA with Dunnett’s multiple comparisons, ***P < 0.001.

Our previous studies showed that a subcutaneous (s.c.) challenge of neonatal C57BL/6 mice with ZIKV (1 × 10^3^ tissue culture ID_50_ [TCID_50_] of ZIKV PRVABC59) leads to a transient viremia followed by infection of the CNS, with viral loads that peak at 9 dpi and then diminish over the following weeks (22) (Figure 1A). ZIKV infects predominantly neurons; causes neuronal apoptosis and neurodegeneration associated with marked cellular infiltration, particularly in the cerebellum, starting on 9 dpi (22); and results in long-term motor and behavioral sequelae (23, 24). Further, acute infection is associated with increased mRNA levels of *Lilrb4* and, to a lesser extent, Pirb paired Ig-like receptor B (*Lilrb3/Pirb*), programmed death ligand 1 (*Pdl1*), and killer cell lectin like receptor C1 (*Klrc1*), that peak at 12–15 dpi and diminish thereafter, mirroring disease progression (Figure 1, B and C). However, the expressions of other LILR genes, such as *Lilra5* and *Lilra6*, or immune checkpoint receptors associated with activated T cells, such as programmed cell death protein 1 (*Pd1*) and cytotoxic T lymphocyte–associated protein 4 (*Ctla4*), were only marginally increased during acute infection despite cellular infiltration (Figure 1B). Flow cytometry analysis showed that LILRB4 expression peaked at 15 dpi and then subsided (Figure 1D). This suggests that LILRB4 could play a pivotal role in regulating the local immune response and limiting the neurological damage induced by ZIKV infection and potentially be a therapeutic target to reduce tissue damage. Importantly, the increase in LILRB4 expression in the brain was also observed in mice challenged with Dengue virus (DENV), Tacaribe virus (TCRV), and Ebola pseudovirus (rVSV-ΔG-EBOV-GP) (Supplemental Figure 1; supplemental material available online with this article; https://doi.org/10.1172/jci.insight.151420DS1), suggesting that LILRB4 plays a role in the response of neonatal mice to neurotropic viruses. Last, flow cytometry analysis showed that during ZIKV infection, LILRB4 was expressed primarily by myeloid cells, mostly microglia (CD45^int^) and infiltrating myeloid cells, including macrophages, neutrophils, and CD11b^+^ DCs (Figure 1, E and F, and Supplemental Figure 2). In contrast, LILBR4 was not expressed on CD45^–^ cells, such as neuronal cells, astroglia, and ependymal and endothelial cells (25) (Figure 1E).

### LILRB4 is critical for survival of ZIKV-challenged mice.

**Figure 2:**
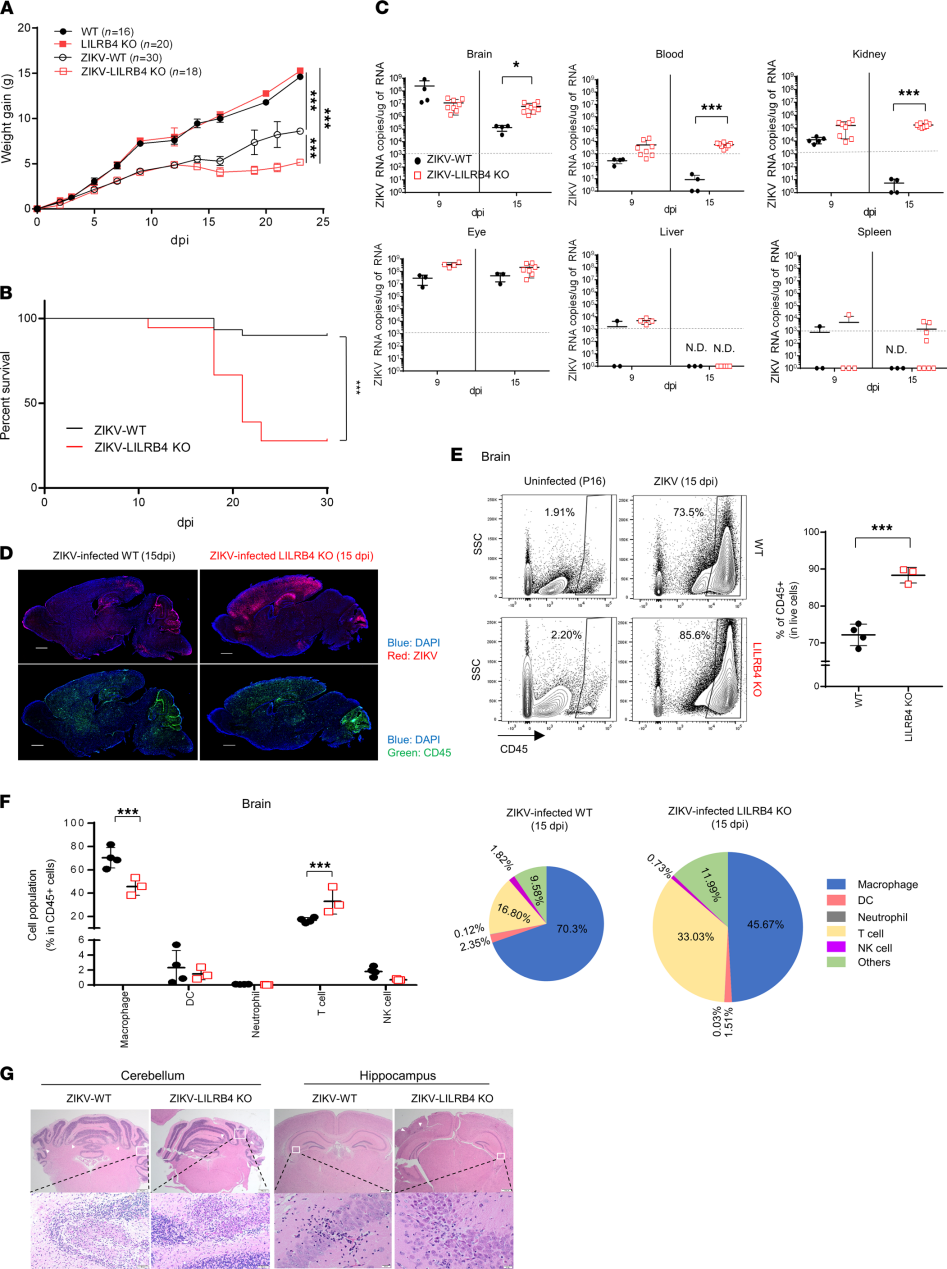
LILRB4 deficiency worsens clinical outcomes in ZIKV infection. (**A** and **B**) P1 WT (*n* = 30) and P1 LILRB4-KO mice (*n* = 18) were challenged with 1000 TCID_50_/mL of ZIKV and monitored for weight changes (**A**) and survival (**B**). Statistical significance was determined by a 2-way ANOVA (**A**) and the log-rank test (**B**), respectively. (**C**) Quantification of ZIKV RNA copies using real-time PCR was performed in ZIKV-infected WT and LILRB4-KO mice at 9 and 15 dpi in the brain, blood, kidney, eye, liver, and spleen (*n* = 3–8 per group). (**D**) Virus and infiltrating cell distribution in the brains of ZIKV-infected WT and LILRB4-KO mice at 15 dpi. The images show representative immunofluorescence staining for CD45 (green), ZIKV (pink), and DAPI (blue) in brain sections from WT and LILRB4-KO mice. (**E** and **F**) Flow cytometry analysis was performed on cells isolated from the brains of uninfected (P16) or ZIKV-infected WT and LILRB4-KO mice at 15 dpi (*n* = 3–4, each group). Live cells were gated and separated based on CD45 expression. The graph shows the percentage of infiltrating cells (CD45^hi^) in total live cells (**E**). CD45^hi^ cells were gated and the population of the following cells was determined: macrophages (CD45^hi^CD11b^+^F4/80^+^), DCs (CD45^hi^CD11c^+^F4/80^–^Ly6G^–^), neutrophils (CD45^hi^CD11b^+^Ly6G^+^F4/80^–^), T cells (CD45^hi^CD3^+^NK1.1^–^), and NK cells (CD45^hi^NK1.1^+^CD3^–^). Both graph and pie charts show the percentage of indicated cells within CD45^hi^ cell population in the brain (**F**). Data were analyzed using 2-tailed unpaired Student’s *t* test (**C**, **E**, and **F**). (**G**) Histopathology of the brains from ZIKV-infected WT and LILRB4-KO mice at 22 dpi. The upper images show representative H&E staining of cerebellum or hippocampus (original magnification, 20×); scale bars: 500 μm. The bottom images show representative brain lesions observed in cerebellum or hippocampus (original magnification, 400×); scale bars: 20 μm. White arrows indicate multifocal malacia or focal gliosis. *P < 0.05, ***P < 0.001. N.D., not detected.

Previous studies suggested that LILRB4 regulates immune cell function in mice infected with vaccinia (14). To determine whether the expression of LILRB4 plays an important role in regulating the susceptibility to infection with ZIKV, LILRB4-KO mice were challenged s.c. with 1 × 10^3^ TCID_50_ of PRVABC59 ZIKV strain on P1. Compared with C57BL/6 WT mice, LILRB4-KO mice had more severe failure to gain weight (Figure 2A) and more pronounced signs of neurological disease, including severe ataxia and paresis/paralysis (Supplemental Figure 3). Last, while most WT mice survived the infection, 72% of LILRB4-KO mice died between 18 and 22 dpi (Figure 2B), indicating that LILRB4 plays a key role in controlling the outcome of ZIKV infection.

To better understand the role of LILRB4, we first examined whether LILRB4-KO mice fail to control the virus. As shown in Figure 2C, the level and tissue distribution of viral RNA observed in WT and LILRB4-KO mice were comparable at 9 dpi, with viral RNA being detectable in the brain, eyes, blood, and kidneys. However, while the levels of viral RNA declined by 15 dpi in WT mice, LILRB4-KO mice showed sustained viral RNA levels, suggesting a failure in viral clearance (Figure 2C). Within the brain the distribution of the virus, predominantly in the cerebellum, cortex, and hippocampus, was similar to that of WT mice, suggesting that LILRB4 deficiency does not change ZIKV tropism (Figure 2D). However, the CNS of LILRB4-KO mice showed a higher number of CD45^hi^ infiltrating cells, particularly in the cortex and hippocampus (Figure 2, D and E). Interestingly, although T cells are thought to mediate viral clearance, the brains of LILRB4-KO mice contained not only increased number of infiltrating CD45^hi^ cells (Figure 2, D and E), but also a relatively higher percentage of T cells (33%) compared with WT mice (16%) at 15 dpi (Figure 2F). Cellular infiltration and gliosis were evident in the cerebellum and hippocampus of infected mice, but the resulting lesions were not more severe in LILRB4-KO than in WT mice (Figure 2G).

### Human and mouse LILRB4 bind ZIKV.

**Figure 3:**
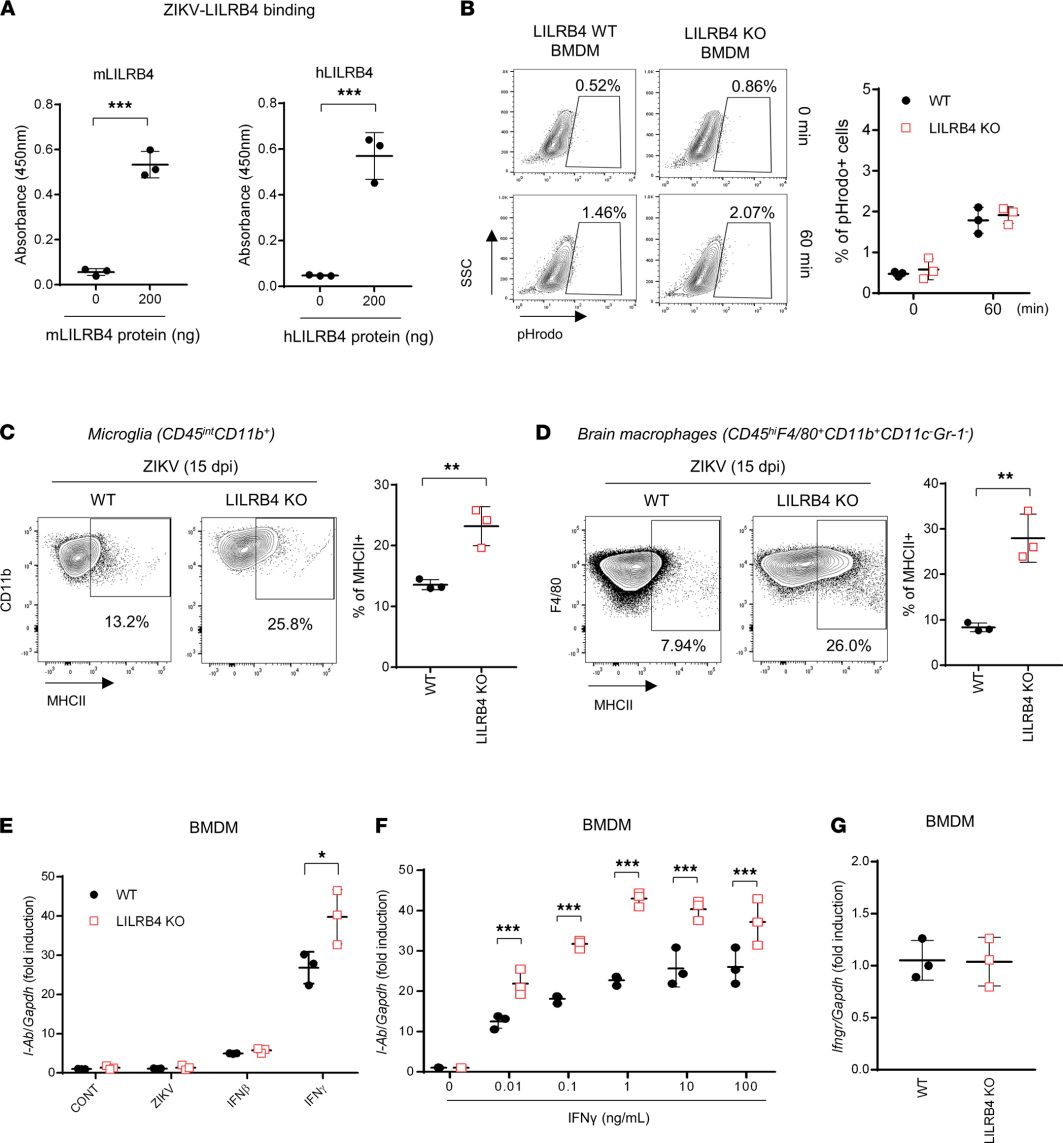
LILRB4 deficiency drives hyperactivation of macrophages and microglia induced by IFN-γ. (**A**) Binding of ZIKV to recombinant mouse LILRB4 (mLILRB4) and human LILRB4 (hLILRB4) proteins. Data are representative of 3 independent experiments (triplicates, each). (**B**) Bone marrow–derived macrophages (BMDMs) from WT and LILRB4-KO mice were infected with pHrodo-stained ZIKV for 0 or 60 minutes in vitro, and cells having internalized ZIKV (pHrodo^+^) were determined by flow cytometry analysis. The plots show the percentage of pHrodo^+^ BMDMs. Data shown as means ± SD of 3 independent experiments. (**C** and **D**) Flow cytometry analysis of MHC class II–positive (MHCII-positive) microglia (**C**) and macrophages (**D**) in the brains of ZIKV-infected WT and LILRB4-KO mice at 15 dpi (*n* = 3, each group). The graphs show the percentage of MHCII^+^ cells in microglia (CD45^int^CD11b^+^) (**C**) and macrophages (CD45^hi^CD11b^+^F4/80^+^) (**D**), respectively. (**E** and **F**) mRNA expression of *I-Ab* on BMDM from WT and LILRB4-KO mice after stimulation with ZIKV (MOI 1), IFN-β (10 ng/mL), or IFN-γ (10 ng/mL) (**E**) or IFN-γ at different doses (**F**) for 24 hours. Data shown as means ± SD of 3 independent experiments. (**G**) mRNA expression of *Ifngr* on BMDMs from WT and LILRB4-KO mice. Data shown as means ± SD (*n* = 3). Data were analyzed using 2-tailed unpaired Student’s *t* test. *P < 0.05, **P < 0.01, ***P < 0.001.

To investigate how LILRB4 helps control ZIKV infection, we first tested whether mouse LILRB4 binds to ZIKV in vitro. As shown in Figure 3A and Supplemental Figure 4A, purified mouse LILRB4 (mLILRB4) proteins, as well as human LILRB4 (hLILRB4) proteins, bind to ZIKV directly in a dose-dependent manner, whereas LILRA5 does not, suggesting that LILRB4 could directly modulate viral infection of cells. However, as shown in Figure 3B, bone marrow–derived macrophages (BMDMs) from naive WT and LILRB4-KO mice were infected with pHrodo-labeled ZIKV to similar degrees, and overexpression of LILRB4 did not enhance ZIKV infection (Supplemental Figure 4B), suggesting that although LILRB4 binds ZIKV, its expression does not directly modulate ZIKV entry or replication.

### LILRB4 regulates IFN-γ–induced macrophage and microglia activation.

Activated microglia and infiltrating macrophages can produce proinflammatory cytokines, glutamate, and reactive oxygen species with the consequent detrimental neuroinflammation described for ZIKV-infected mice (22, 26). LILRB4 is known to regulate the activation of myeloid cells (10, 17, 27). As shown in Figure 3, C and D, microglia and infiltrating macrophages in the brains of ZIKV-infected LILRB4-KO mice showed increased expression of MHC class II (MHCII), a marker of activation, relative to age-matched infected WT mice, suggesting that LILRB4 plays a role in regulating their activation during ZIKV meningoencephalitis.

To determine whether the increased MHCII expression on microglia and macrophages in these animals reflects the higher viral load or an intrinsic defect in the regulation of the cell’s activation state, we examined their response to IFN-γ, a major mediator of microglial/macrophage activation ex vivo (28, 29). Of note, upon ZIKV infection, mice lacking IFN-γ failed to upregulate MHCII expression (Supplemental Figure 5). As shown in Figure 3E, an increased number of BMDMs derived from naive LILRB4-KO mice upregulated MHCII in response to IFN-γ as compared with those of WT mice. The effect was specific and not evident in cells stimulated with the virus or IFN-β. A dose-response curve showed that IFN-γ induced higher MHCII expression in LILRB4-deficient BMDMs than in those from WT mice, and the maximal level of expression was higher in the absence of LILRB4 (Figure 3F) despite the similar levels of *Ifngr* mRNA expression (Figure 3G), indicating that LILRB4 expression increases the cell activation threshold. Together, these data suggest that myeloid cells in LILRB4 KO have an intrinsic defect in their response to IFN-γ that could underlie the severe symptomatology, and their increased activation level is not merely a reflection of the increased viral load in the CNS.

### Impact of LILRB4 on adaptive immune cell responses.

**Figure 4:**
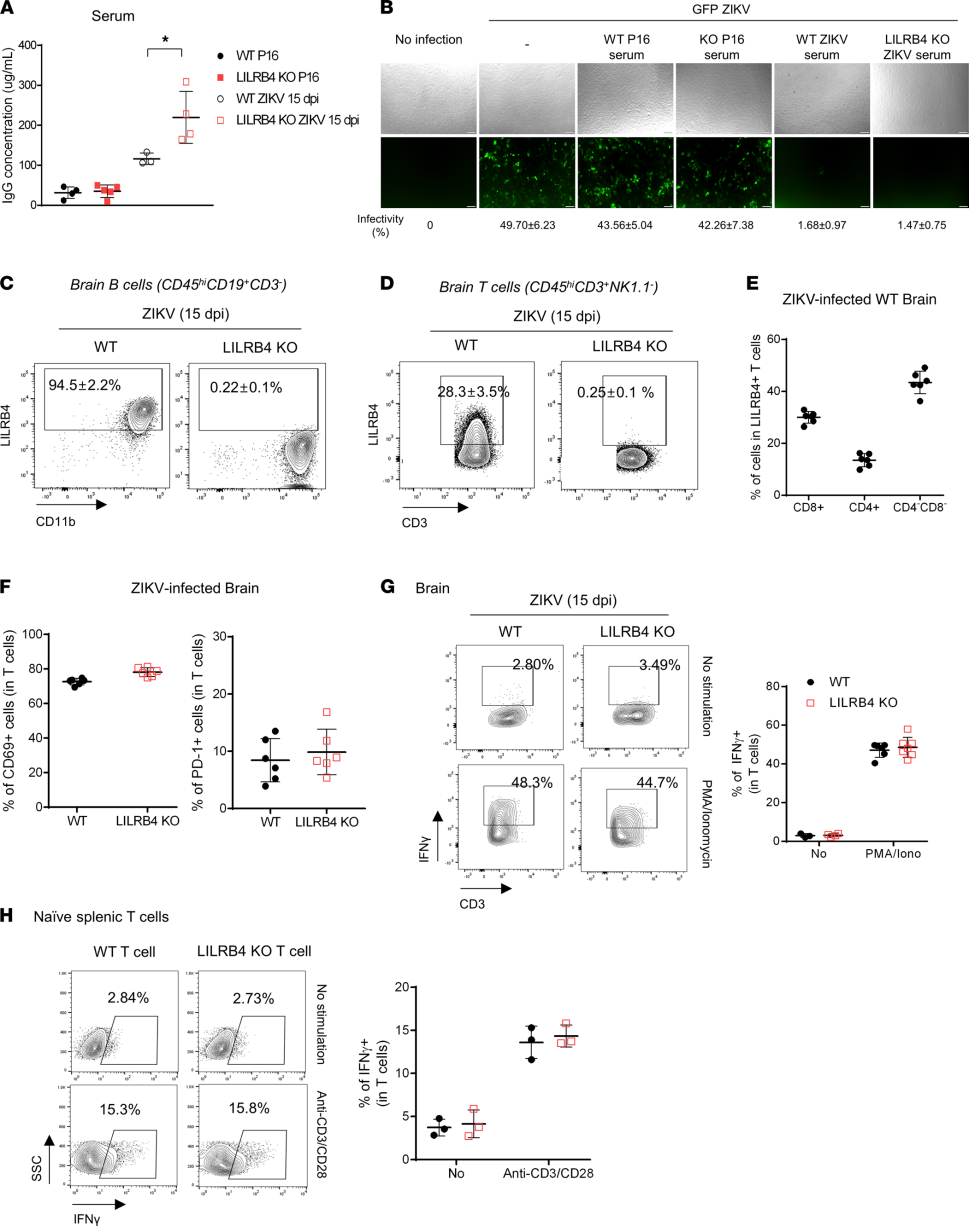
LILRB4 expression on B and T cells is not a determining factor in protecting mice from ZIKV-induced death. B and T cells’ responses were evaluated in WT and LILRB4-KO mice at 15 dpi. (**A**) IgG levels in sera were measured by ELISA (*n* = 3–5, each group). (**B**) Neutralization assay using Vero E6 cells infected with ZIKV-GFP in the presence of sera (1:10 dilution). The percentage of infectivity was determined using ImageJ (NIH). Upper panel: Bright-field. Lower panel: GFP expression. Scale bar: 100 μm. (**C** and **D**) The percentages of LILRB4-expressing B cells (CD45^hi^CD19^+^CD3^–^) (**C**) and T cells (CD45^hi^CD3^+^NK1.1^–^) (**D**) were determined by flow cytometry in the brains of ZIKV-infected WT and LILRB4-KO mice. Data shown as mean ± SD of 6 mice per group. (**E**) LILRB4-expressing T cells characterized by CD4 and CD8 expression. (**F**) CD69 and PD-1 staining of T cells infiltrating the brain. (**G** and **H**) IFN-γ expression in T cells isolated from the brains of ZIKV-infected WT and LILRB4-KO mice (**G**) and in naive splenic T cells stimulated with anti-CD3/CD28 for 24 hours (**H**). Data are representative of 3 independent experiments. Left: Representative flow data of 3 independent experiments. Right: Percentage of IFN-γ^+^ in T cells. Data were analyzed using 2-tailed unpaired Student’s *t* test. *P < 0.05.

Neutralizing antibodies and activated T cells are thought to be critical for ZIKV clearance (30–33). LILRB4 is expressed on activated and memory B cells and helps regulate their Ig production, and mice lacking LILRB4 make higher levels of antibodies (15). Next, we determined whether the lack of LILRB4 is associated with a paradoxical impaired humoral response in our model. As shown in Figure 4A, LILRB4-KO mice mounted higher levels of IgG antibodies against the virus at 15 dpi compared with WT mice, supporting that LILRB4 plays a role in controlling antibody production. Further, we showed that the antibodies neutralized ZIKV infectivity in vitro (Figure 4B). This suggests that LILRB4 expression by B cells is not required to mount an antibody response to ZIKV, and the failure to control the virus evident after 12 dpi is not due to a defect in the humoral response.

We next examined lymphocytes infiltrating the CNS for LILRB4 expression. As shown in Supplemental Figure 6A, while naive resting lymphocytes did not express LILRB4, about 12.6% of naive T cells upregulated LILRB4 upon activation with anti-CD3/CD28 along with CD69 and CD44. Among activated splenic T cells, LILRB4 was primarily expressed by CD8^+^ T cells (Supplemental Figure 6B). However, LILRB4 expression was not required for T cell activation as CD8^+^ T cells from WT and LILRB4-KO mice showed similar levels of CD62L and CD44 (Supplemental Figure 6C). In vivo, over 95% of B cells infiltrating the brains of ZIKV-infected WT mice at 15 dpi, which were mostly CD11b^+^, expressed LILRB4 (Figure 4C); in contrast, only 28% of CD45^hi^CD3^+^NK1.1^–^ T cells expressed LILRB4 (Figure 4D). Of the latter, 30% were CD8^+^ T cells, and 13.4% were CD4^+^ T cells, but most of them were double-negative T cells (Figure 4E). T cells infiltrating the brains of mice lacking LILRB4 showed similar levels of expression of activation markers (CD69 and PD-1) as WT mice (Figure 4F), indicating that LILRB4 expression is not necessary for T cell activation. Further, despite a previous report showing an increase in IFN-γ production during vaccinia infection in mice lacking LILRB4 (14), the number of T cells from ZIKV-infected brains producing IFN-γ ex vivo or upon stimulation with PMA/ionomycin was not increased in LILRB4-KO mice (Figure 4G). Similarly, lack of LILRB4 expression did not increase IFN-γ production among CD8^+^ T cells isolated from the infected brains (Supplemental Figure 7). Last, to confirm the role of LILRB4 in regulating IFN-γ production by T cells in vitro, we isolated splenic T cells from naive WT or LILRB4-KO mice and stimulated them with anti-CD3/CD28 antibodies (Figure 4H). As with the T cells derived from the CNS, the proportion of IFN-γ–producing T cells in the spleen was similar for both strains upon stimulation with anti-CD3/CD28 antibodies. Taken all together, our findings indicate that defects in B and T cells are unlikely to underlie the reduced viral clearance in LILRB4-KO mice.

### Altered NK cells underlie the severe ZIKV infection in LILRB4-deficient mice.

Although the number of NK cells infiltrating the CNS was lower than that of T cells (Figure 2F), they can play a key role in viral clearance by killing infected cells through exocytosis of cytolytic granules containing perforin and granzyme, or by secreting proinflammatory cytokines, in particular IFN-γ, and are therefore considered the first line of defense against viral infection such as DENV and Sindbis virus (34–36). Using antibodies against NK1.1 to deplete NK cells in vivo, we confirmed that these cells play a critical role in protection against meningoencephalitis by ZIKV, as mice lacking NK cells had higher viral load in the brain and increased mortality (Supplemental Figure 8).

**Figure 5:**
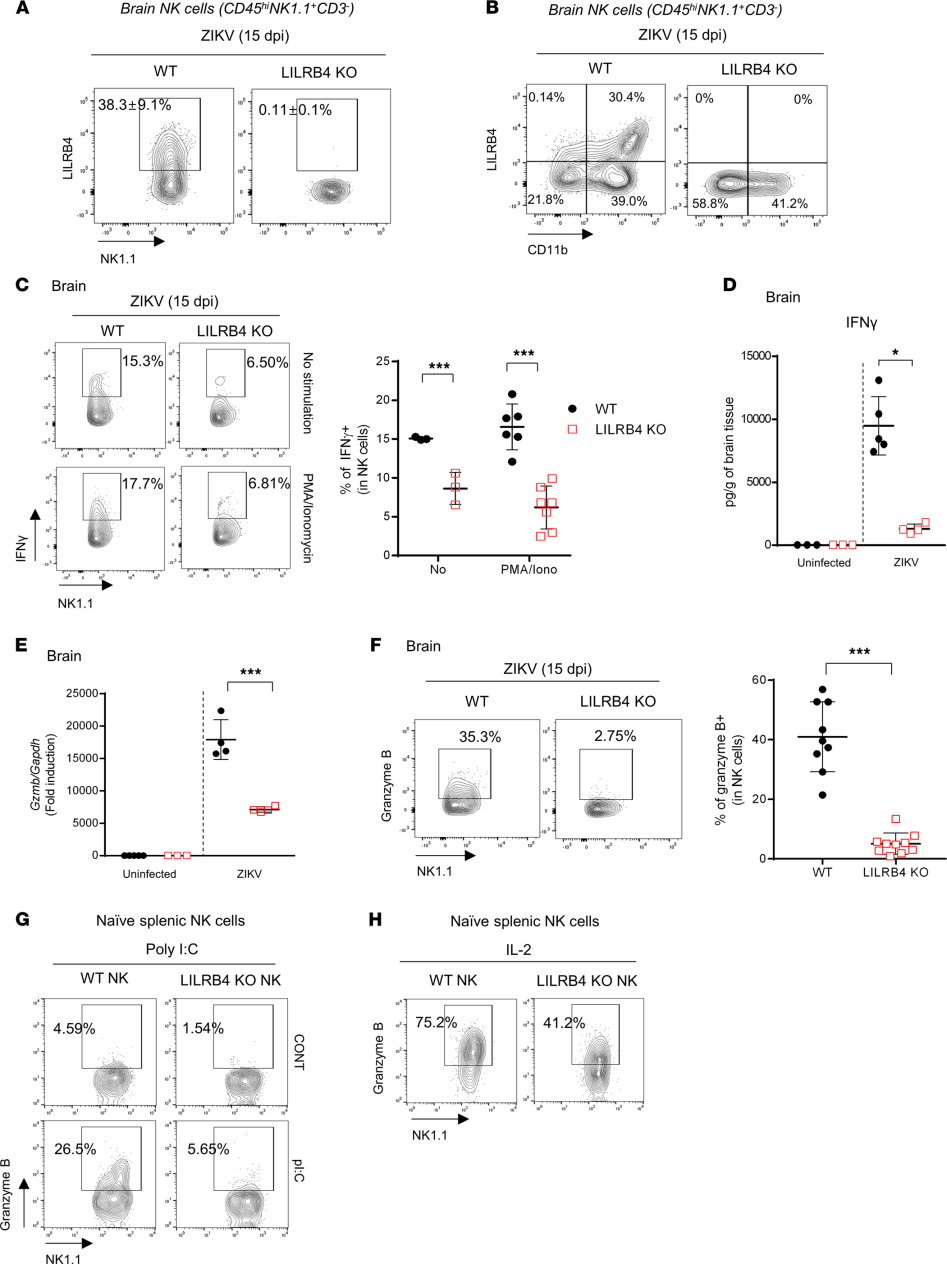
LILRB4 deficiency leads to defects in NK cell activation. (**A** and **B**) Percentage of LILRB4-expressing (**A**) and CD11b-expressing (**B**) NK cells (CD45^hi^NK1.1^+^CD3^–^) in the brains of ZIKV-infected mice at 15 dpi. Data shown as the mean ± SD (*n* = 6 mice/group). (**C**) IFN-γ expression in brain NK cells. Left: Representative flow data. Right: The percentage of IFN-γ^+^ in NK cells. (**D**) IFN-γ levels in the brain (Luminex; *n* = 3–6/group). (**E**) *Gzmb* mRNA levels in the brain. Mean ± SD (*n* = 3–4/group). (**F**) Granzyme B expression in NK cells from the brains of ZIKV-infected mice. (**G** and **H**) Granzyme B expression in naive splenic NK cells stimulated with poly(I:C) (100 μg/mL) for 24 hours (**G**) or IL-2 (50 ng/mL) for 48 hours (**H**). Data are representative of 3 independent experiments, each performed in triplicate or quadruplicate. Data were analyzed using 2-tailed unpaired Student’s *t* test. *P < 0.05, ***P < 0.001.

As with lymphocytes, naive NK cells did not express LILRB4 (Supplemental Figure 9A) but could upregulate it upon activation. In ZIKV-infected mice, 38.3% of the NK cells (mostly CD11b^+^ NK cells) that infiltrated the brain displayed LILRB4 expression (Figure 5, A and B). To examine whether LILRB4 deficiency affected the function of NK cells infiltrating the infected brain tissue, we first examined their IFN-γ production ex vivo. As shown in Figure 5C, 15% of NK cells infiltrating the CNS of infected WT mice produced IFN-γ. Unlike T cells (Figure 4F), NK cells from the brains of LILRB4-KO mice showed reduced IFN-γ production (Figure 5C), and this resulted in an overall reduction in IFN-γ levels in the brains of ZIKV-infected LILRB4-KO mice as compared with WT (Figure 5D). The impairment was not limited to NK cells infiltrating the CNS, as a relative reduction in IFN-γ production was also evident in splenic NK cells from ZIKV-infected LILRB4-KO mice stimulated with PMA/ionomycin (Supplemental Figure 10A). In addition to IFN-γ, fully mature NK cells accumulated granzyme B in their secretory lysosomes that is critical for their cytolytic function, and its production increases during infection. As shown in Figure 5E, the mRNA levels for *Gzmb* in the brains of infected LILRB4-KO mice were significantly reduced compared with infected WT mice. Intracellular staining confirmed that granzyme B expression in NK cells was markedly reduced in the brain and spleen of ZIKV-infected LILRB4-KO mice compared with WT mice (Figure 5F and Supplemental Figure 10B). The defect in granzyme B production was also evident upon stimulation of naive splenic NK cells with IL-2 or poly(I:C) (Figure 5, G and H), suggesting an intrinsic NK cell deficit in the absence of LILRB4.

**Figure 6:**
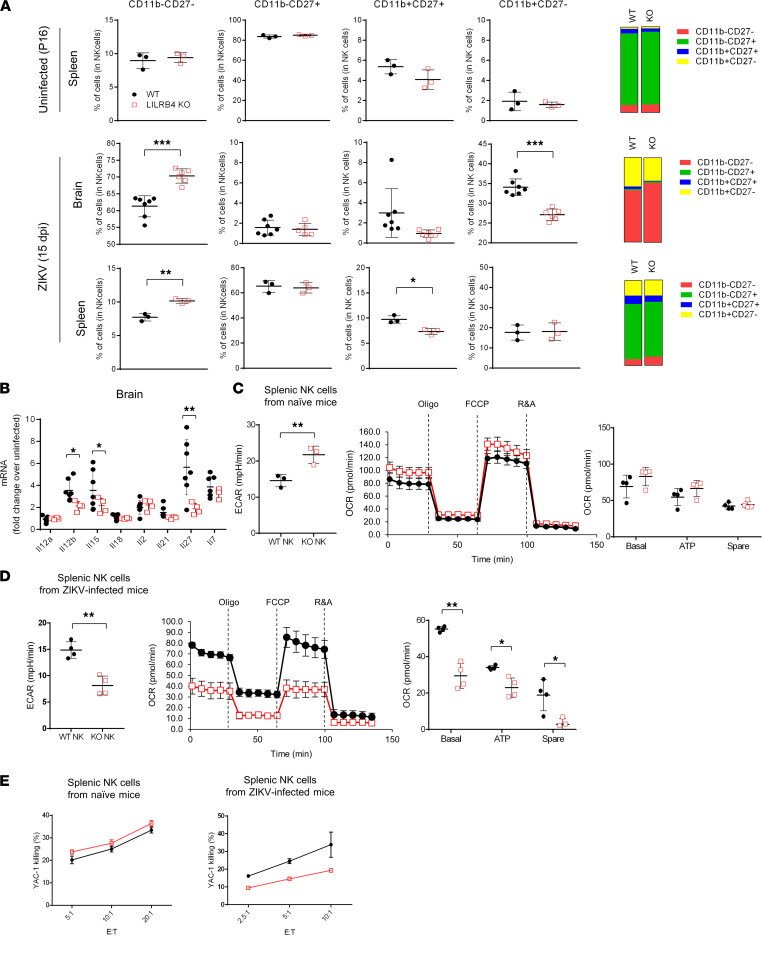
LILRB4 deficiency causes changes in NK cell maturation and metabolism during ZIKV infection. (**A**) NK cell maturation determined by CD27 and CD11b staining. The stacked bar graphs show the percentage of NK cell population based on CD27 and CD11b expression. (**B**) RNA expression of *Il-12*α, *Il-12*β, *Il-15*, *Il-18*, *Il-2*, *Il-21*, *Il-27*, and *Il-7* in the brains of ZIKV-infected mice at 15 dpi. Data presented as fold induction over age-matched uninfected controls for each strain. (**C**–**E**) NK cells were isolated from pooled spleens of naive (P16; *n* = 5 per strain) or ZIKV-infected (15 dpi; *n* = 10–16 per strain) mice. ECAR and real-time changes of OCR were measured in IL-2–stimulated NK cells from naive mice (**C**) or nonstimulated NK cells from ZIKV-infected mice (**D**). Bar graphs indicate basal respiration (Basal), ATP production (ATP), and spare respiratory capacity (Spare). Cytotoxicity assay was performed against YAC-1 target cells. The graphs summarize the percentage of 7-aminoactinomycin D–positive (7-AAD^+^) YAC-1 cells (**E**). Data are representative of 3 independent experiments, each performed in triplicate or quadruplicate. Data were analyzed using 2-tailed unpaired Student’s *t* test. *P < 0.05, **P < 0.01, ***P < 0.001.

The reduced levels of IFN-γ and granzyme B observed suggest that LILRB4 deficiency could impair development or activation of NK cells. In mice, changes in expression of CD11b and CD27 reflect the progressive differentiation and tissue residence of NK cells and have been used to stage NK cell development (37, 38). The expression of CD27 and CD11b on NK cells in the spleen of WT and LILRB4-KO mice at P16 showed a similar distribution of NK cell subpopulations (Figure 6A). In contrast, the spleens of infected LILRB4-KO mice showed a relative increase in immature CD11b^–^CD27^–^ NK cells and a relative decrease in effector CD11b^+^CD27^+^ NK cells (Figure 6A). Among NK cells that localized to the brain, ZIKV-infected LILRB4-KO mice had a significant reduction in CD11b^+^CD27^–^ fully mature NK cells compared with WT mice (Figure 6A). Moreover, these phenotypic changes were associated with relatively lower mRNA levels in infected brains of LILRB4-KO mice for IL-12, IL-15, IL-21, and IL-27 (Figure 6B), which are thought to play a role in the commitment and development of NK cells (39–41). Together, these suggest an impaired ability of NK cells from LILRB4-KO mice to respond to infection.

The capacity of NK cells to respond to a virus may be dictated by their metabolic status (42, 43). To investigate whether changes in NK cell subpopulations in infected LILRB4-KO mice are associated with metabolic changes, we measured extracellular acidification rate (ECAR) and oxygen consumption rate (OCR), indicative of glycolysis and mitochondrial respiration, respectively. As shown in Figure 6C, IL-2–stimulated splenic NK cells from naive LILRB4-KO mice had a higher ECAR than those from naive WT mice, suggesting a higher glycolytic rate. The mitochondrial respiration, as shown by the basal respiration rate, ATP production, and spare respiration capacity, was comparable in NK cells from naive LILRB4-KO mice and naive WT mice (Figure 6C). These data suggest that LILRB4 does not significantly affect the metabolism of IL-2–stimulated splenic NK cells from naive mice. In contrast, when NK cells were harvested from the spleens of infected animals, NK cells from LILRB4-KO had reduced ECAR compared with those from WT mice (Figure 6D), suggesting a reduced glycolytic capability. Their mitochondrial respiration was also decreased, with a 50% decrease in basal respiration rate, a 30% decrease in ATP production, and a loss of their spare respiration capacities (Figure 6D). These data suggest that NK cells from infected LILRB4-KO mice present an overall silenced metabolism with restricted metabolic flexibility. In addition, the lack of spare respiration capacity might affect how NK cells from infected LILRB4-KO mice adapt to changes in their environment.

Intact metabolic pathways are critical for NK cytolysis and viral clearance (43). Consistent with the above findings, IL-2–stimulated splenic NK cells derived from naive WT and LILRB4-KO mice showed similar killing of YAC-1 cells (Figure 6E). In contrast, NK cells derived from infected LILRB4-KO mice showed reduced cytotoxic activity as compared with those of infected WT mice (Figure 6E). Together, these data suggest that impaired NK cell function could underlie the reduced viral clearance and increased death observed in LILRB4-KO mice.

**Figure 7:**
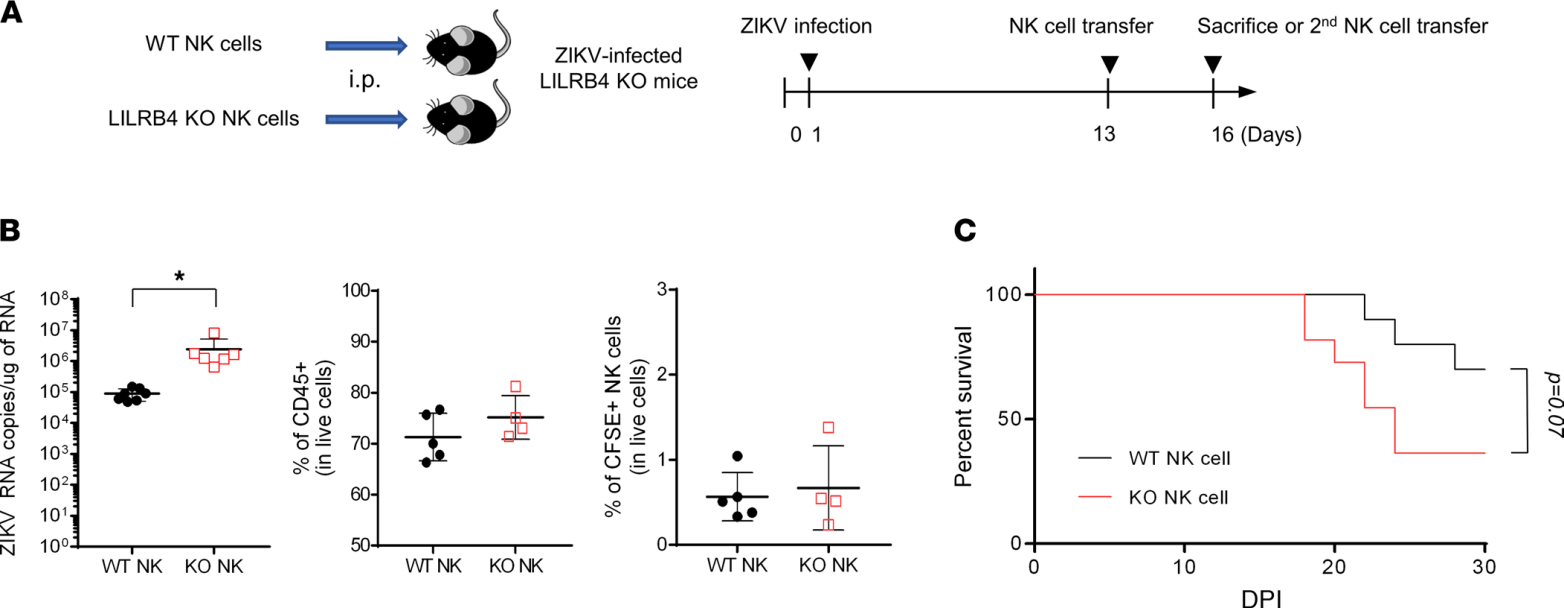
LILRB4 deficiency in NK cells contributes to impaired viral clearance and ZIKV-induced death. (**A**) Purified, CFSE-labeled NK cells (3 × 10^5^ cells) derived from naive WT or LILRB4-KO mice were injected i.p. into ZIKV-infected LILRB4-KO mice at 12 dpi. Three days later (15 dpi), mice were sacrificed to measure viral RNA in brain or received a second NK cell transfer and were followed to assess survival. (**B**) At 15 dpi, ZIKV RNA copies were quantified using real-time PCR from the brain, and the percentage of CD45^hi^ cells and transferred NK cells (CFSE^+^CD45^+^NK1.1^+^) in the brain was determined by flow cytometry analysis. Data are representative of 2 independent experiments. Data were analyzed using 2-tailed unpaired Student’s *t* test. *P < 0.05. (**C**) Survival of ZIKV-infected LILRB4-KO mice (*n* = 10 for WT NK cell transfer and *n* = 11 for LILRB4-KO NK cell transfer) that received purified NK cells (3 × 10^5^ cells i.p.) derived from naive WT or LILRB4-KO mice at 12 and 15 dpi. Statistical significance was determined by 2-tailed unpaired Student’s *t* test and the log-rank test.

To verify that impaired NK cell function in LILRB4-KO mice is sufficient to reduce viral clearance and survival, we next transferred purified, CFSE-labeled splenic NK cells from naive WT or LILRB4-KO mice into LILRB4-KO mice that had been challenged with ZIKV 12 days before (Figure 7A). Three days after transfer (15 dpi), CFSE-labeled NK cells were detected in the brain, indicating that NK cells from WT and LILRB4-KO mice similarly homed to the infected brain (Figure 7B). Of note, mice that received NK cells from WT mice showed reduced CD45^hi^ cell infiltration and viral load compared with those that received NK cells derived from LILRB4-KO mice (Figure 7B). In addition, the LILRB4-KO mice that received NK cells from WT animals showed improved survival compared with those that received NK cells from LILRB4-KO mice (Figure 7C). Together, our findings indicate that LILRB4 plays a critical role in the maturation and activation of NK cells that localize to the brain and that while NK cells from mice lacking LILRB4 expression appear functionally intact in the resting state, their functional deficiency becomes evident under the stress of an infection.

## Discussion

Our study describes a biological role for LILRB4 as a mediator of effective NK cell maturation and activation during a viral infection in mice. We show that acute encephalitis by multiple viruses, including ZIKV, leads to an increased number of cells expressing LILRB4 in the brain that are critical to controlling the infection, as mice lacking LILRB4 fail to clear the virus in the CNS, develop more severe neurologic symptoms, and eventually succumb to the disease. In the infected brain, LILRB4 is expressed by activated microglia as well as most infiltrating myeloid cells, B cells, and a fraction of activated T cells and CD11b^+^ mature NK cells. Interestingly, although LILRB4 is a checkpoint receptor bearing 2 ITIMs, and known to downregulate the activation of antigen-presenting cells, B cells, and T cells, this study suggests that the more severe outcome in LILRB4-KO mice is due to altered NK cell maturation and function leading to diminished control of viral load and further increases in mortality. Indeed, adoptive transfer of WT NK cells was sufficient to improve viral clearance and survival of ZIKV-infected LILRB4-KO mice. Interestingly, while naive NK cells from LILRB4-KO mice had reduced IFN-γ and granzyme B production upon stimulation but did not show phenotypic, metabolic, or cytolytic differences from the ones from WT mice, NK cells retrieved from infected LILRB4-KO mice failed to produce granzyme B and IFN-γ and had reduced glucose and oxygen consumption and lower cytolytic capacity. This suggests that LILRB4 expression is required for NK cells to sustain an effective response to infection.

Upregulation of LILRB4 expression in the brain was described in microglia of aged mice as well as surrounding the plaques in a mouse model of Alzheimer’s disease, and its expression was associated with an immune suppressive/tolerizing function (44, 45). More recently, in patients infected with SARS-CoV-2, LILRB4 levels in peripheral blood have been correlated with disease progression (46). Increased LILRB4 expression in microglia is consistent with previous data from Waschbisch et al. suggesting that LILRB4 is induced by type I IFNs (47), which are upregulated early in the infectious process and critical to contain the viral expansion. In addition, cellular infiltration by myeloid cells and activated lymphocytes during infection contributes to the increased LILRB4 levels in meningoencephalitis.

Modeling studies of protein-protein interactions examining human protein binding to ZIKV suggested that ZIKV proteins have the potential to bind human LILRAs and LILRBs (48). Further, LILRB1 was previously reported to bind another flavivirus, DENV2 (49). However, it was unclear whether ZIKV binds LILRB4 in biological systems. Our data show that mouse and human LILRB4 bind ZIKV particles directly. Further, the binding is selective, as we also found that ZIKV can bind to human LILRB4 and LILRB1 but not LILRA5 (Supplemental Figure 4A). Importantly, their absence does not appear to modify virus entry in LILRB4-expressing myeloid cells (Figure 3B) despite resulting in increased levels of Src homology region 2 domain-containing phosphatase 1 (SHP-1) phosphorylation (Supplemental Figure 11), suggesting that the role of LILRB4 role in viral infection is linked to their immunomodulatory activity and not to increased viral entry. Since human LILRB4 expression is distinct from mouse LILRB4 despite their similar function, whether LILRB4 plays a similar protective role against ZIKV in humans as it does in mice will need to be investigated. LILRB4 is expressed on antigen-presenting cells and monocytic leukemia cells in both humans and mice (50); however, unlike mouse LILRB4, human LILRB4 is not expressed on conventional T and NK cells (51). Moreover, while integrin α_v_β_3_ is a well-known ligand of mouse LILRB4, this does not bind to human LILRB4, and the ligand of human LILRB4 is still unknown (51). Nevertheless, this is probably the first paper suggesting the virus as the ligand for human and mouse LILRB4 and suggesting a clinical relevance for the mouse system in investigating the role of LILRB4 in controlling viral infection in humans.

It is known that LILRB4 regulates immune cell activation and mediates their suppressive activity via a long cytoplasmic domain that contains ITIMs (9). Consistent with this, our studies show that BMDMs from LILRB4-KO mice had a conserved antigen uptake and presentation in vitro (Supplemental Figure 12) but a magnified MHCII expression upon stimulation with IFN-γ. Despite this, in vivo, infected LILRB4-KO mice did not show increased levels of proinflammatory cytokines in the CNS including IL-1α and IL-1β or antiinflammatory cytokine IL-10 (Supplemental Figure 13), while TNF-α and IL-6 levels were modestly lower, and their lesions were not significantly worse than those in WT animals. Lower levels of TNF-α and IL-6 might be due to upregulation of SHP-1 phosphorylation, which mediates the inhibition of proinflammatory cytokine production (52) in LILRB4-deficient cells (Supplemental Figure 11). All together, these findings suggest that the impaired survival in LILRB4-KO mice does not result from excessive inflammation.

Previous studies have shown that neonatal mice are susceptible to infection with ZIKV while adult WT mice are resistant (22). The mechanisms underlying the increased susceptibility to neurotropism in young mice are unknown but may be rooted in the high frequency of mitotically active immature neurons and neuronal precursors, active pruning, and differential expression pattern of cellular receptors used by the virus to gain entry to cells (53, 54). Here we show that in the absence of LILRB4 signaling, the progression of infection resembled that of WT mice in terms of viral expansion in the CNS, neurological symptoms, and brain lesions early in the disease. However, these mice failed to reduce the viral load in the CNS, and the infection was lethal. This suggests that the early immune response to the virus, presumably by type I IFN, is conserved in the absence of LILRB4. Instead, the reduction in viral clearance and survival appears to be tied to reduced NK cell function.

Several studies have described lymphocytic infiltration in the brain and the critical role of T cells in ZIKV encephalitis (30, 55), including memory CD4^+^ T cells required to generate virus-specific humoral responses (31). Paradoxically, in LILRB4-KO mice, the increased viral load evident by 15 dpi was associ¬ated with a relative increase in the number of infiltrating lymphocytes in the CNS, with no evidence of an impaired adaptive response since the antibody titers in LILRB4-KO mice were more robust than those of WT animals, and cytokine production by T cells appeared largely conserved. Further, the percentage of CD3^+^ T cells expressing IFN-γ spontaneously ex vivo or capable of producing IFN-γ upon stimulation was similar to that of WT mice. This finding suggests that lack of LILRB4 does not alter T cell function significantly, although Gu et al. have demonstrated upregulation of IFN-γ production by T cells during vaccinia infection in LILRB4-KO mice (14). This discrepancy could be due to neonatal T cells being less responsive than adult T cells, therefore increasing susceptibility to infection (56). Alternatively, it is possible that the apparent minimal effect of LILRB4 on T cell activation and IFN-γ production in our model could be linked to its expression in only 30% of T cells infiltrating the infected brain, and mostly on CD4^–^CD8^–^ double-negative T cells. While double-negative T cells infiltrating the brain have been described in ischemic brain tissues of both stroke patients and middle cerebral artery occlusion mice and are associated with local neuroinflammation (57), their relative contribution to the overall IFN-γ and granzyme response is unknown. Together, our data suggest that neither T nor B cell defects underlie the reduced control of viral replication or the increased mortality in infected LILRB4-KO mice.

NK cells control a variety of viral infections through IFN-γ secretion and induction of antigen-nonspecific death of infected cells (58). Their central role in controlling ZIKV infection was confirmed by the increased viral loads and reduced survival in mice treated with anti–NK1.1 antibodies and the rescue in ZIKV-infected LILRB4-KO mice that received NK cells from WT mice. Their activity is regulated by multiple inhibitory receptors, including Ly49, killer IgG receptors, and T cell immunoreceptor with Ig and ITIM domains (TIGIT) that regulate NK cell maturation and function (59, 60). In ZIKV-infected mice, 30%–40% of NK cells that infiltrated the brains express LILRB4, and they mostly corresponded to mature CD11b^+^ NK cells, which are the most effective in cytokine secretion and cytotoxic capacity. Remarkably, NK cells from the spleens of uninfected LILRB4-KO mice showed similar levels of maturation as determined by CD11b and CD27 staging. These findings are consistent with those published by Rojo et al. showing no significant impairment in NK cell function in naive LILRB4-KO mice (61). In our hands, however, even NK cells from uninfected LILRB4-KO mice showed shifts in the inhibitory receptor CD94/NK group 2 member A and Ly49C/I/F/H expression, indicating altered maturation (62) (Supplemental Figure 9B), as well as a modest reduction of granzyme B that became more evident upon stimulation with poly(I:C) or IL-2. This suggests that LILRB4 deficiency in NK cells is associated with inherent NK cell functional defects. Further, NK cells from naive LILRB4-KO mice showed slightly increased cytotoxic activity and glucose consumption as compared with those from naive WT mice. Interestingly, the defects became more evident in NK cells embedded in the brains of infected LILRB4-KO mice, as they included a higher frequency of immature CD11b^–^ phenotype and showed a significant reduction in IFN-γ and granzyme B production as compared with those of WT mice. Moreover, assessment of their metabolic and cytolytic capacity revealed a marked reduction in glucose/oxygen consumption and target cell killing in LILRB4-deficient mice following infection. The reduced cytotoxicity and lower glycolysis we observed is consistent with previous studies suggesting that naive and immature NK cells rely on glucose fueled by oxidative phosphorylation for their cytotoxic activity whereas educated blood-derived NK cells have increased rates of glycolysis that enable them to clear virus more efficiently (63).

The reduced activity of NK cells in the context of an ongoing infection could be attributed to exhaustion. However, NK cells from infected LILRB4-KO mice showed impaired production of IFN-γ and granzyme B even at 9 dpi and more importantly did not upregulate other receptors linked to exhaustion, such as PD-1 (Supplemental Figure 14). Alternatively, the absence of LILRB4 could result in altered maturation. It has been reported that inhibitory receptors such as Ly49 and TIGIT contribute to NK cell education, a process for acquiring functional maturation and self-tolerance, and NK cells lacking those inhibitory receptors show developmental defects and poor cytotoxicity with less IFN-γ production (64–66). LILRB4 is a regulatory receptor that is expressed on NK cells only upon activation and has not been implicated previously in NK cell maturation and activation. However, LILRB4 expression is inducible on microglia, monocytes, DCs, and other immune cell types and could contribute to the regulation of immune microenvironment that determines the maturation of NK cells in the periphery. It is therefore possible that the defect in NK cells is secondary to a shift in the immune milieu during development. The mechanisms by which the defect in NK cells in LILRB4-KO mice becomes more severe during infection are also unknown. CD11b^–^ immature NK cells compose most of the NK cell population in neonatal mice while adult mice have mostly CD11b^+^ NK cells at peripheral sites including peripheral blood and spleen (37). It is possible that in our neonatal ZIKV mouse model, more of those immature NK cells migrated to the inflamed tissue or that NK cells that migrated to the infected tissue cannot fully mature in the absence of LILRB4. However, the marked reduction in granzyme B production by naive NK cells and even more profound functional defects in NK cells within the infected tissue indicate impairment beyond the relative reduction in CD11b^+^CD27^–^ NK cells.

Inhibitory immune checkpoint receptors expressed on activated immune cells trigger immunosuppressive pathways that are critical for limiting tissue damage resulting from the response to the virus, but they may play additional roles that are critical for controlling infections (67). For example, upon murine coronavirus strain 3 infection, PD-1 is highly expressed on T cells, NK cells, and macrophages, and its deficiency is associated with overproduction of IFN-γ and TNF-α, which may enhance viral clearance, but also with increased fibrinogen deposition, worse hepatitis, and higher mortality compared with WT mice (5). Similarly, LILRB3 (PirB in mice) deficiency or antibody blockade causes increased inflammation, with higher levels of IL-6 and TNF-α and lower levels of IL-10 (68). Interestingly, despite increased TNF-α and nitric oxide production, these mice also have inefficient phagosomal oxidation that results in reduced clearance of salmonella compared with WT mice, indicating that LILRB3 plays a role in immune responses beyond that of controlling excessive responses (69). Consistent with this dual effect, our studies show that LILRB4 deficiency exaggerates BMDM responses to IFN-γ and antibody production upon infection but also results in defects in NK cell function that impair viral clearance and may hinder tissue repair. These findings are consistent with the recent description of dual inhibitory and activating functions, depending on the position of the functional tyrosine residues in its ITIMs and/or the nature of the stimuli (70). Thus, while checkpoint receptors like LILRB4 can be effectively targeted to treat some cancers or revert exhausted CD8^+^ T cells from mice chronically infected with LCMV (71), their role in acute infection is complex and should be gauged carefully when using therapeutically.

To our knowledge, this is the first paper demonstrating the importance of NK cells in controlling ZIKV infection. More importantly, we establish that LILRB4 plays a key role in NK cell maturation and function. While the absence of LILRB4 signaling appears to minimally affect NK cells of healthy mice, during infection fewer NK cells attain a mature phenotype, resulting in impaired cytolytic function. This highlights a previously unappreciated function of LILRB4 in NK cell development and maturation that may help improve the efficacy of therapies for infectious diseases.

## Methods

### Mice.

C57BL/6 (WT) mice were purchased from the Jackson Laboratory, and *gp49b^–/–^* (LILRB4-KO) mice on the C57BL/6 background were provided by Eric Long (National Institute of Allergy and Infectious Diseases/NIH, Rockville, Maryland, USA) (61). WT and LILRB4-KO mice were bred and housed in the specific pathogen–free, Association for Assessment and Accreditation of Laboratory Animal Care International–accredited animal facility of the FDA’s Division of Veterinary Medicine (Silver Spring, Maryland, USA). In all in vivo experiments, newborns were used. This study was carried out in strict accordance with the recommendations in the Public Health Service Policy on Humane Care and Use of Laboratory Animals (Office of Laboratory Animal Welfare, 2015).

### ZIKV infection.

ZIKV PRVABC59 (Puerto Rico strain), isolated by the CDC (described in our previous publications: refs. 22, 72), was used in this study. All pups were born from naive parents and inoculated at P1 with 1000 TCID_50_ of ZIKV by s.c. injection. For experiments tracking survival following ZIKV infection, mice were monitored daily for clinical signs of pathology and weighed every other day to minimize handling. All procedures were performed in accordance with the FDA Animal Care and Use Committee guidelines.

### ZIKV quantification.

Mice were euthanized by CO_2_ asphyxiation and exsanguinated by transcardiac perfusion. Tissues were removed aseptically, placed in 1 mL of TRIzol reagent (Invitrogen), and disrupted with Precellys 24 homogenizer (Bertin Technologies) until uniform homogenates were obtained. ZIKV RNA levels were measured using quantitative one-step reverse transcriptase (Promega) PCR to amplify ZIKV genome position 1087 to 1163 (GenBank accession no. AY632535). We used 1 μg of isolated total RNA for each sample, and ZIKV RNA levels are expressed as ZIKV copies/μg of total RNA using a standard curve, as previously described (22).

### Real-time PCR.

Total RNA was extracted from BMDMs or brain tissues using TRIzol. After DNase treatment (TURBO DNA-*free* Kit ; Thermo Fisher Scientific), 1 μg of total RNA was used for cDNA synthesis using using High-Capacity cDNA Reverse Transcription Kit (Thermo Fisher Scientific) per manufacturer’s protocol. Gene expression assays were performed using a Viia7 real-time PCR machine with QuantStudio software (both from Thermo Fisher Scientific). Gene fold change in expression (ΔΔCt) was determined by normalizing Ct values to GAPDH (housekeeping gene) and then to uninfected or untreated controls.

### Expression analysis.

mRNA expression levels of immune genes were determined using the NanoString nCounter gene expression system (NanoString Technologies). Isolated RNA (100 ng) was hybridized with probes from nCounter Mouse Inflammation v2 Panel at 65°C for 18 hours. Afterward hybridized products were prepared for cartridge loading on an nCounter PrepStation. Digital counting of fluorescent signals was conducted using the nCounter Digital Analyzer. Data analysis including statistics was carried out with the nSolver4.0 software (NanoString Technologies).

### Flow cytometry.

Brain tissues were collected after an intracardiac perfusion with sterile PBS and digested in RPMI 1640 containing 0.1% trypsin plus 0.015% DNase I for 30 minutes at 37°C with pipetting every 10 minutes. Cells from brains were resuspended in 10 mL of 30% Percoll (GE Healthcare, now Cytiva) and then underlaid with 1 mL of 70% Percoll. After centrifugation at 800*g* at 4ºC for 30 minutes, cells were collected from the 30%–70% interface, washed in RPMI 1640, and isolated by centrifugation at 400*g* at 4ºC for 5 minutes. This method is optimized for collecting microglia and infiltrating mononuclear cells. Splenocytes were obtained by dissociating the spleen into single-cell suspensions using a 70 μm cell strainer. Cells were resuspended in ACK lysis buffer to remove red blood cells. Isolated cells were stained with a cocktail of antibodies against various lineage markers, including CD45 (BioLegend, 103149), F4/80 (BioLegend, 123120), CD11b (BioLegend, 101242), CD11c (BioLegend, 117318), Ly6G (BioLegend, 127633), CD3 (BioLegend, 100351), NK1.1 (Invitrogen, 12-5941-82), CD4 (BD Biosciences, 552775), CD8a (BioLegend, 100747), and gp49 (BioLegend, 144906). To check IFN-γ or granzyme B expression, cells were incubated with brefeldin A (10 μg/mL) with or without PMA (10 ng/mL) and ionomycin (1 μg/mL) for 3.5 hours. For intracellular staining, cells were fixed with 2% paraformaldehyde for 12 minutes on ice, permeabilized using 0.5% saponin in PBS, and stained with antibodies specific to IFN-γ (BD Biosciences, 554411) or granzyme B (BioLegend, 515405) for 1 hour on ice. Flow cytometry data were collected with the LSRFortessa X-20 flow cytometer (BD Biosciences), and the results were analyzed using FlowJo software (version 10).

### Histopathology.

Tissues were obtained from ZIKV-infected WT or LILRB4-KO mice at 22 dpi and fixed in 10% phosphate-buffered formalin. Samples were embedded in paraffin, sectioned, and stained with hematoxylin and eosin. Histopathology was evaluated blindly. Evaluation included heart, spleen, liver, kidney, and brain. However, significant lesions were only noted in the brain, so these are the data presented.

### Immunofluorescence analysis.

Brains were collected and fixed as described (22). Tissues were stained with anti-4G2 (MilliporeSigma, MAB10216) or anti-CD45 antibodies (BD Biosciences, 550539). Sections were imaged using a Panoramic Digital Slide Scanner (3DHistech). Images were captured using Panoramic Viewer software (3DHistech). All images were prepared for publication using Adobe Photoshop CC 2015 software.

### Preparation of BMDMs.

BM cells were isolated from femurs and tibias of WT and LILRB4-KO mice. BMDM differentiation was induced by culturing of BM cells in RPMI 1640 medium supplemented with 10% FBS and 30% LADMAC cell-conditioned medium (a source of M-CSF) for 7 days with 1 addition of fresh culture medium containing 30% LADMAC cell-conditioned medium on day 3. On day 7, after removal of nonadherent cells, BMDMs were detached from the plate using Accutase (Innovative Cell Technologies).

### ELISA.

Immulon 2 HB flat-bottom plates (96-well; Thermo Fisher Scientific) were coated with 200 μL of 1 × 10^6^ TCID_50_/mL of ZIKV in Opti-MEM I medium (Gibco). For ZIKV protein binding assay, plates were incubated with recombinant mLILRB4 or hLILRB4 proteins, followed by primary antibodies against target proteins overnight at 4°C. For antibody titer assay, plates were incubated with serum samples overnight. The signals were read with VICTOR X Light Luminescence Plate Reader (PerkinElmer) at 450 nm.

### ZIKV internalization assay.

ZIKV PRVABC59 was labeled with pHrodo (Invitrogen), then isolated using Mag4C-LV magnetic nanoparticles (OZ Biosciences). BMDMs from WT and LILRB4-KO mice were infected with pHrodo-labeled ZIKV (MOI 1) for 60 minutes, followed by suspension in an alkaline buffer (pH 8.8) to quench pHrodo fluorescence signal from extracellular ZIKV. ZIKV-infected cells (pHrodo^+^) were determined by flow cytometry analysis using the FACSCalibur (BD Biosciences), and the results were analyzed using FlowJo software (version 10).

### Neutralization assay.

Vero E6 cells purchased from ATCC were seeded at 3 × 10^4^ cells/well in 96-well plates. ZIKV-GFP (1 × 10^5^ TCID_50_) was incubated with sera (1:10 dilution) from uninfected or ZIKV-infected WT and LILRB4-KO mice (drawn at 15 dpi) for 1 hour at 37°C, then overlaid on top of the Vero E6 cell monolayers. After incubating for 48 hours at 37°C, GFP-positive cells were detected using an Olympus VS-120 Virtual Microscope.

### Extracellular flux and ATP assays.

Extracellular flux assays were performed using an XFe96 Analyzer (Agilent) according to the manufacturer’s instructions. Briefly, 96-well flux plates were coated with 20 μL of poly-l-lysine (MilliporeSigma), and NK cells were adhered to plates in a non-CO_2_ chamber at 37°C for 1 hour prior to the assay. NK cells were analyzed in triplicate in nonbuffered XF base medium supplemented with 2 mM l-glutamine, glucose to a final concentration of 10 mM, and 1 mM sodium pyruvate. ECAR results shown represent the average over 30 minutes. OCR was measured under basal conditions, followed by the injections of oligomycin (1 μM), FCCP (1 μM), and rotenone/antimycin A (500 nM). This allows the accurate calculation of oxygen consumption due to basal respiration, spare respiration capacity, and ATP production. Nonmitochondrial oxygen consumption = minimum rate measurement after rotenone/antimycin A injection; basal respiration = last rate measurement before oligomycin injection – nonmitochondrial oxygen consumption; maximal respiration = maximum rate measurement after FCCP injection – nonmitochondrial oxygen consumption; spare respiratory capacity = maximal respiration – basal respiration; ATP production = last rate measurement before oligomycin injection – minimum rate measurement after oligomycin injection.

### Cytotoxicity assay.

Splenic NK cells were isolated using EasySep Mouse NK Cell Isolation Kit (StemCell Technologies), then incubated with IL-2 (50 ng/mL) for 48 hours. Splenic NK cells from ZIKV-infected mice were purified using EasySep Mouse NK Cell Isolation Kit, then directly used for cytotoxicity assay. NK cells (effector cells, E) were mixed with YAC-1 target cells (T; provided by Venkateswara Simhadri, FDA, Silver Spring, Maryland, USA) that were labeled with 5 μM of CFSE in the indicated E/T ratio. After 4 hours of incubation at 37°C, cells were stained with 7-AAD. Results are shown as a mean percentage of 7-AAD^+^ in CFSE^+^ cells ± SD.

### Adoptive transfer of NK cells.

CFSE-labeled NK cells (3 × 10^5^ cells) were injected i.p. into ZIKV-infected LILRB4-KO mice at 12 dpi. Brain tissues were collected from the mice at 15 dpi. For survival studies, LILRB4-KO mice were s.c. injected with 1000 TCID_50_ of ZIKV at P1, then i.p. injected with NK cells (3 × 10^5^ cells) at 12 and 15 dpi. Mice were observed for over 30 days.

### Statistics.

Weight gain was analyzed by a 2-way ANOVA. Survival experiments were analyzed by the log-rank test. Two-tailed unpaired Student’s *t* test or 1-way ANOVA with Dunnett’s multiple comparisons were used as appropriate; *P* values were adjusted for multiple comparisons as appropriate. Statistical analyses were conducted with GraphPad Software (version 7.03). *P* < 0.05 was considered statistically significant.

### Study approval.

All protocols involving animals were approved by the Animal Care and Use Committee at the FDA (protocol number 2019-02).

## Author contributions

HNL, MM, and DV designed the study; HNL mainly performed the experiments and analyzed the data; APL, KE, MC, and DDCI helped with the experiments; MG performed the extracellular flux and ATP assays; MAE evaluated histopathology; HNL, MM, CS, and DV discussed the data; HNL wrote the manuscript; DV revised the manuscript.

## Supplementary Material

Supplemental data

## Version 1 02/08/2022

Electronic publication
