# Supplementary material for: NK cells require immune checkpoint receptor LILRB4/gp49B to control neurotropic Zika virus infections in mice
Source: JCI Insight. 2022 Feb 8;7(3):e151420. doi: 10.1172/jci.insight.151420 (PMC8855830; doi:10.1172/jci.insight.151420)
Supplement: Supplemental data [file jciinsight-7-151420-s067.pdf]

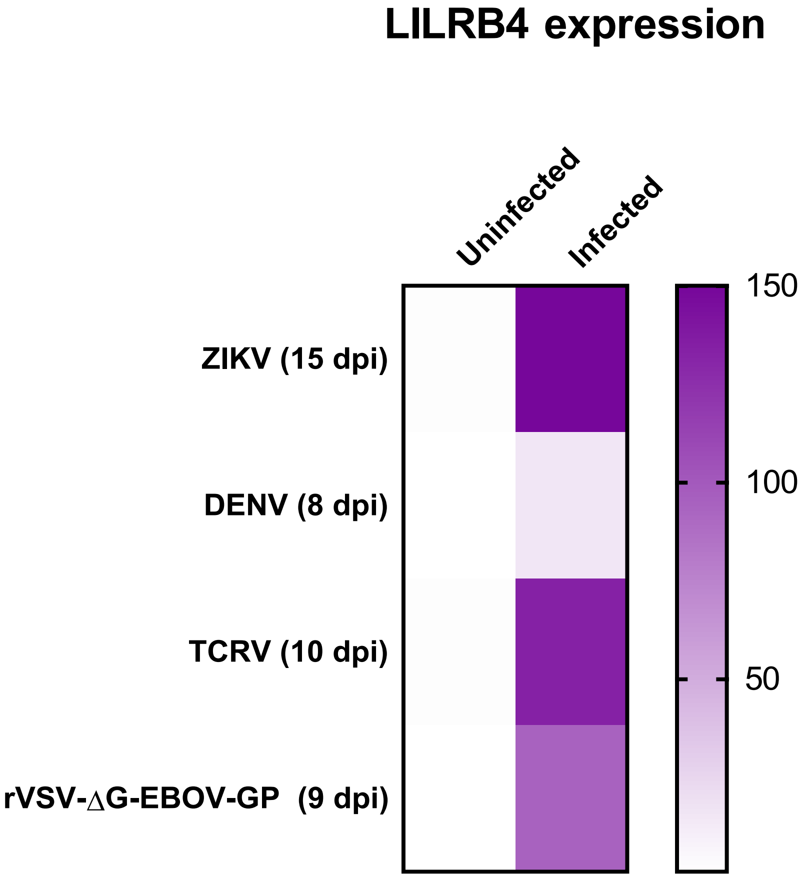

Supplemental Figure 1. LILRB4 expression in the brain of ZIKV, DENV, TCRV and rVSV-ΔG-EBOV-GP-infected mice. Heat map shows the fold changes in LILRB4 RNA expression in the brain of ZIKV (15 dpi), DENV (8 dpi), TCRV (10 dpi) and rVSV-ΔG-EBOV-GP (9 dpi)-infected mice relative to uninfected mice with age matched. RNA expression was assessed by NanoString analysis using the nCounter mouse Immunology panel.

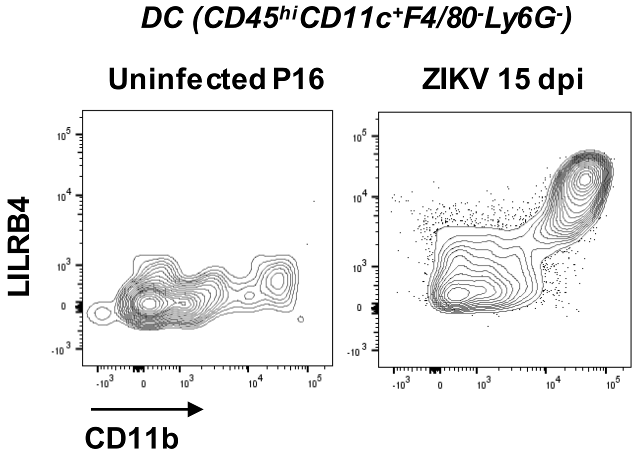

Supplemental Figure 2. LILRB4 expression is detected on CD11b$^+$ DC in the mouse brain during ZIKV infection. The phenotype of LILRB4-expressing DCs (CD45^{hi}CD11c$^+$F4/80$^-$Ly6G$^-$) was determined by flow cytometry in the brain of uninfected (P16) and ZIKV-infected WT mice at 15 dpi. DCs (CD45^{hi}CD11c$^+$F4/80$^-$Ly6G$^-$) were gated and separated based on CD11b and LILRB4 staining. Data are representative of two independent experiments (n=3-5, each time point).

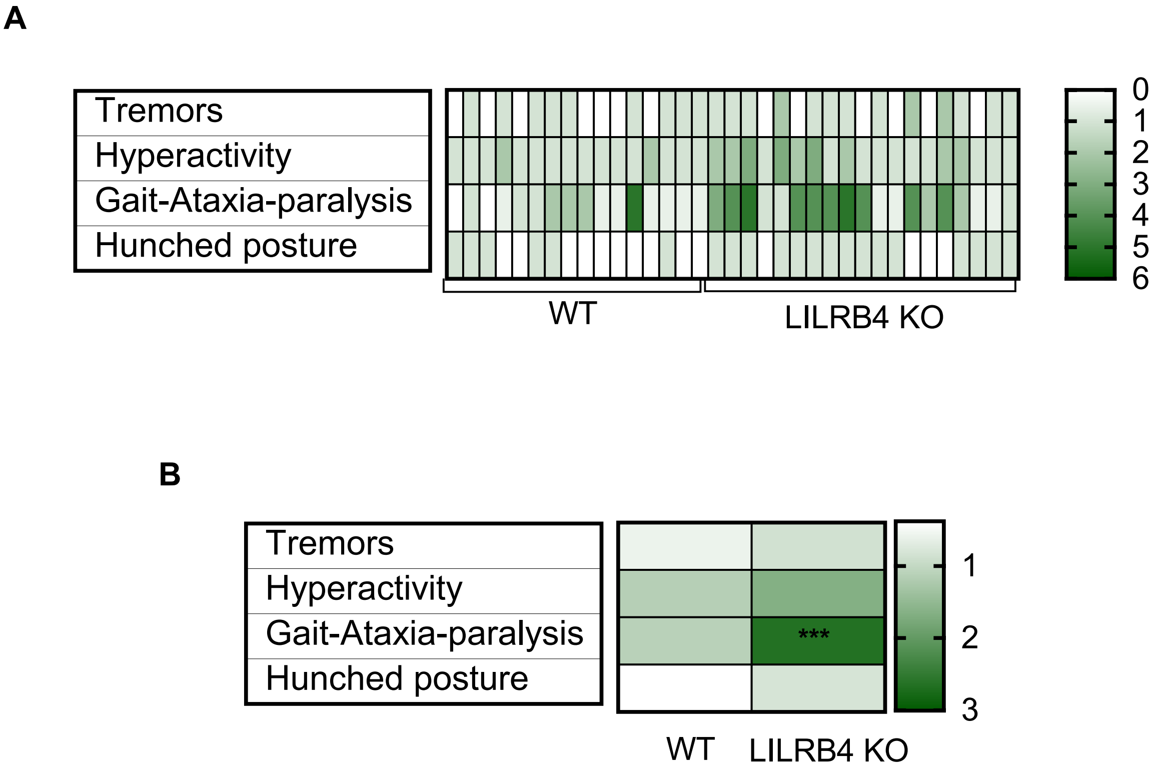

Supplemental Figure 3. Clinical symptoms in ZIKV-infected WT and LILRB4 KO mice at 15 dpi. (A and B) Neurological symptoms were determined in ZIKV-infected WT (n=16) and LILRB4 KO mice (n=19) at 15 dpi and scored as follows: Tremors were graded 0 for none, 1 for occasional, 2 for low but constant and 3 for rapid and constant. Hyperactivity was scored 0 for normal, 1 for skittish, 2 for mouse running laps in the cage and 3 for mouse running into walls. Gait-Ataxia-Paralysis was scored 0 of normal, 0.5 for wide stance, 1 for wide stance and poor balance, 2 for gait-neuropathy, 3 for paresis, 4 for paralysis in one leg and 5 for paralysis in both legs. Hunched posture was graded 0 for normal, 1 for present and 2 for if posture impedes normal function. Heat maps visualize clinical scores for individuals **(A)** or the mean of a group **(B)**. ***$P < 0.001$

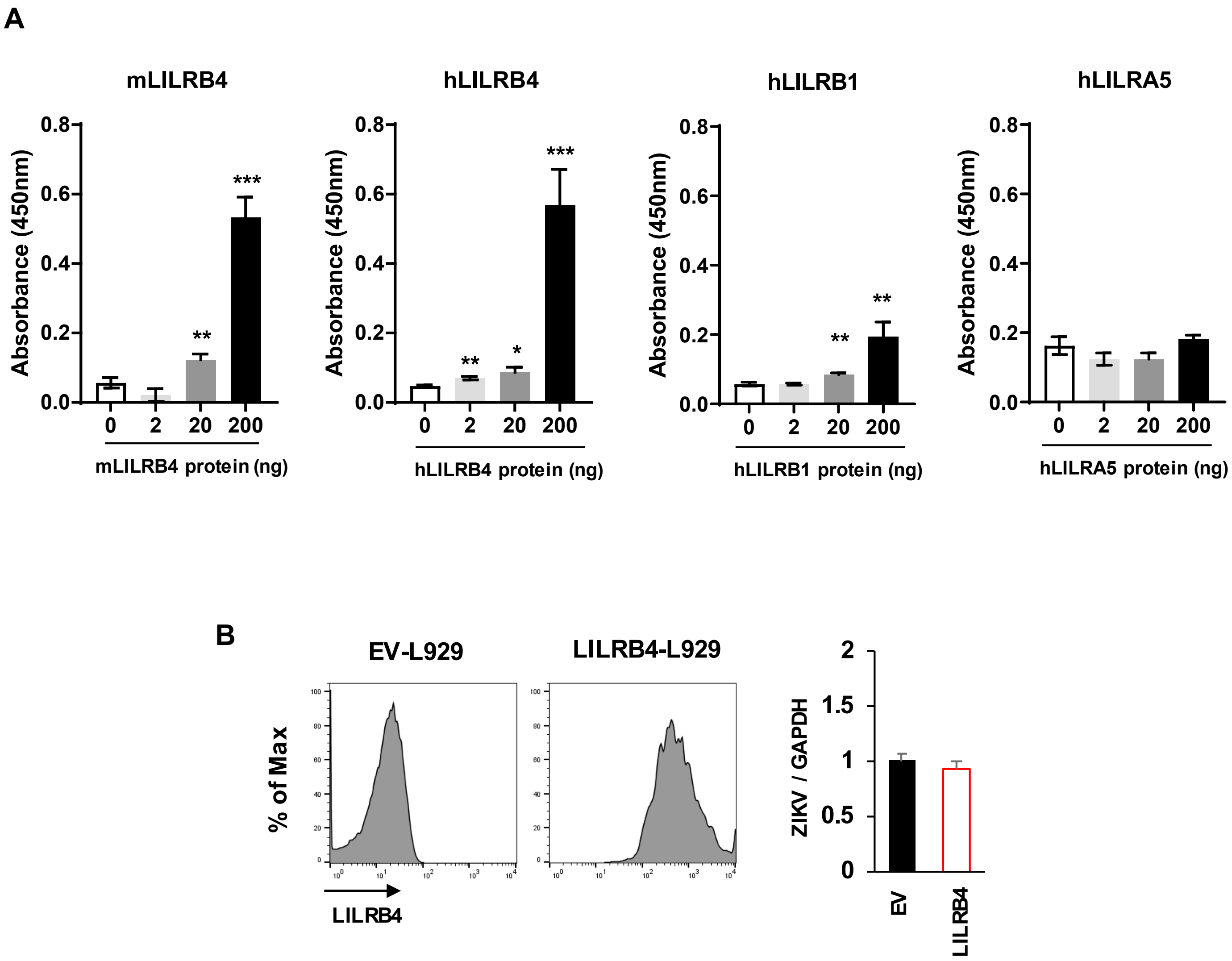

Supplemental Figure 4. LILRB4 binds to ZIKV but does not promote ZIKV infection. (A) Binding of ZIKV to mLILRB4, hLILRB4, hLILRB1 and hLILRA5 recombinant proteins. Data are representative of three independent experiments (triplicates, each). *$P < 0.05$, **$P < 0.01$, ***$P < 0.001$. **(B)** Mouse fibroblast L929 cells, transduced with empty virus (EV) or lentivirus encoding LILRB4, were infected with ZIKV (MOI 1) for 24 h. Relative quantification of ZIKV RNA copies was using Real-time PCR was used to determine relative ZIKV RNA levels normalized with GAPDH. Data shown as means + S.D of three independent experiments.

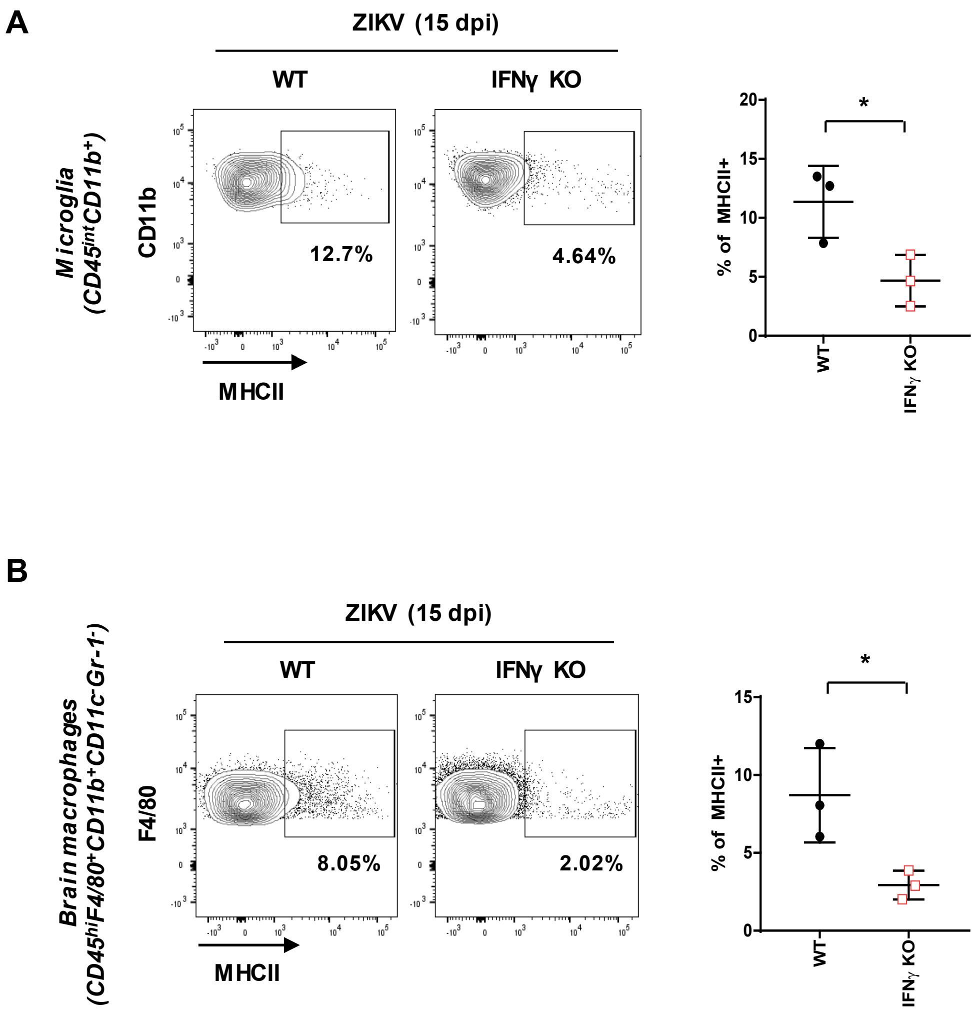

Supplemental Figure 5. **Impaired activation of microglia and brain macrophages in ZIKV-infected IFNγ KO mice.** Flow cytometry analysis of MHCII-positive microglia **(A)** and macrophages **(B)** in the brain of ZIKV-infected WT and IFNγ KO mice at 15 dpi. The graphs show the percentage of $MHCII^{+}$ cells in microglia ($CD45^{int}CD11b^{+}$) **(A)** and macrophages ($CD45^{hi}CD11b^{+}F4/80^{+}$) **(B)**, respectively.

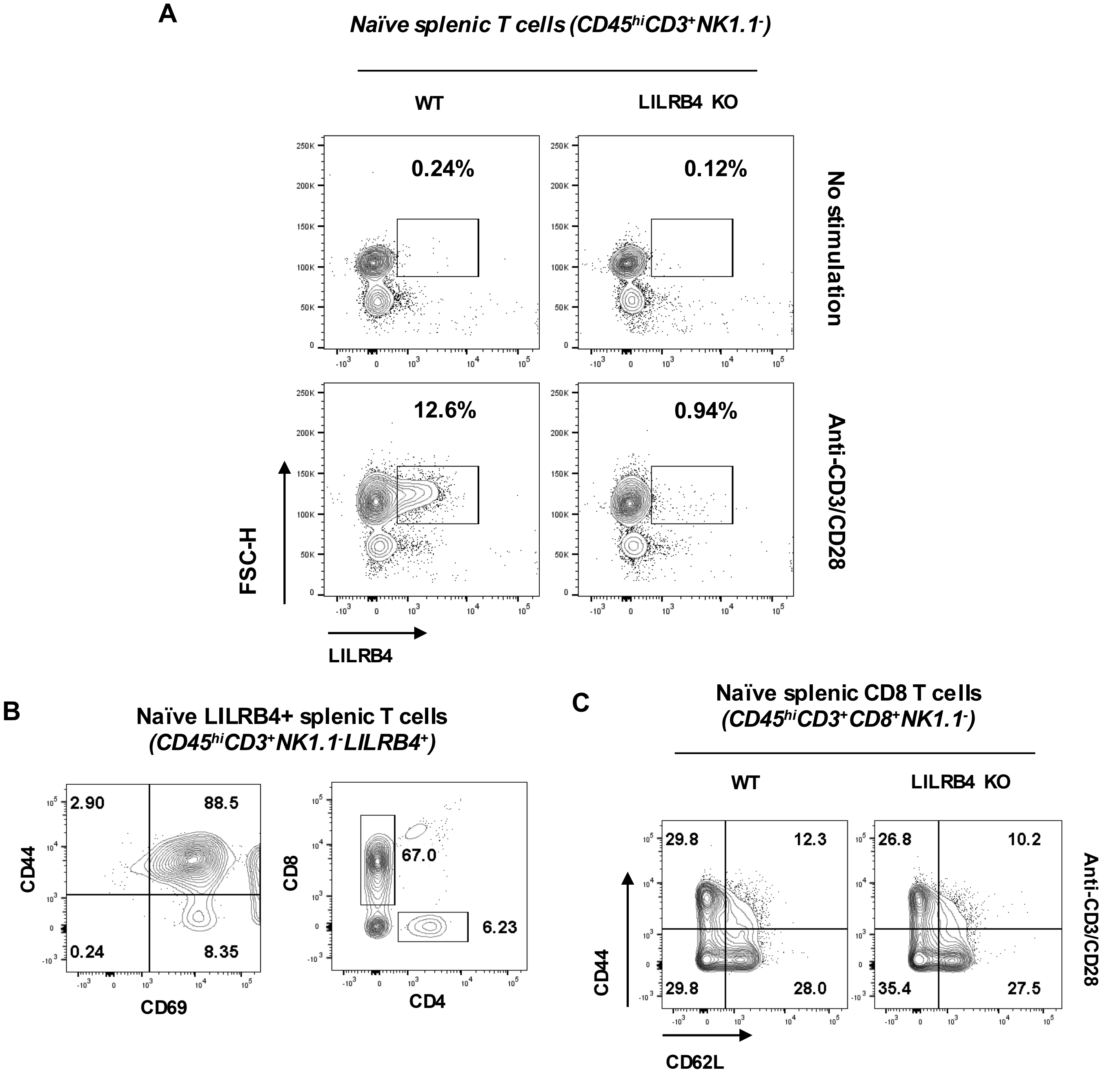

Supplemental Figure 6. Activated CD8 splenic T cells express LILRB4. (A-C) T cells were isolated from the spleen of naïve WT and LILRB4 KO mice at P16, and then stimulated with Dynabeads mouse T-Activator CD3/CD28 for 24 h. T cells ($CD45^+CD3^+NK1.1^-$) were separated based on LILRB4 expression. The plots show representative results of the percentage of LILRB4-expressing cells in T cells **(A)**. T cell subtypes were determined by CD44, CD62L, CD4 and CD8 staining within LILRB4$^+$ T cells **(B)**. T cell phenotype was determined by CD44 and CD62L expression within CD8+ T cells **(C)**. Data are representative of two independent experiments.

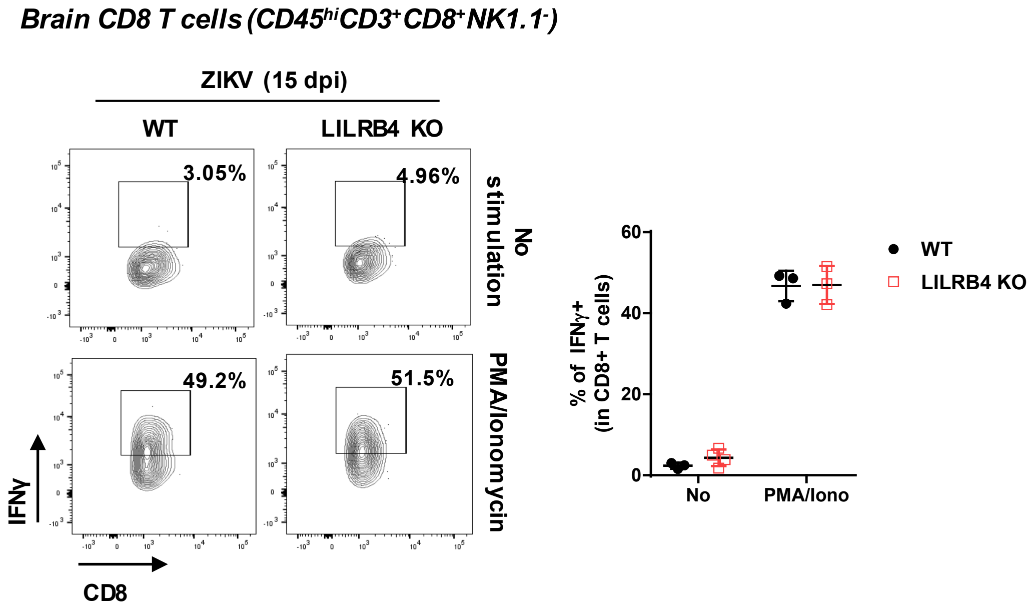

Supplemental Figure 7. LILRB4 is not critical for regulating IFNγ production by CD8 T cell during ZIKV infection. Cells were isolated from the brain of ZIKV-infected WT and LILRB4 KO mice at 15 dpi, and then incubated with brefeldin A (10 µg/mL) with or without PMA (10 ng/mL) and ionomycin (1 µg/mL) for 3.5 h. The intracellular expression of IFNγ was determined by flow cytometry in CD8 T cells ($CD45^{hi}CD3^{+}CD8^{+}NK1.1^{-}$). The graphs show the percentages of CD8 T cells expressing IFNγ.

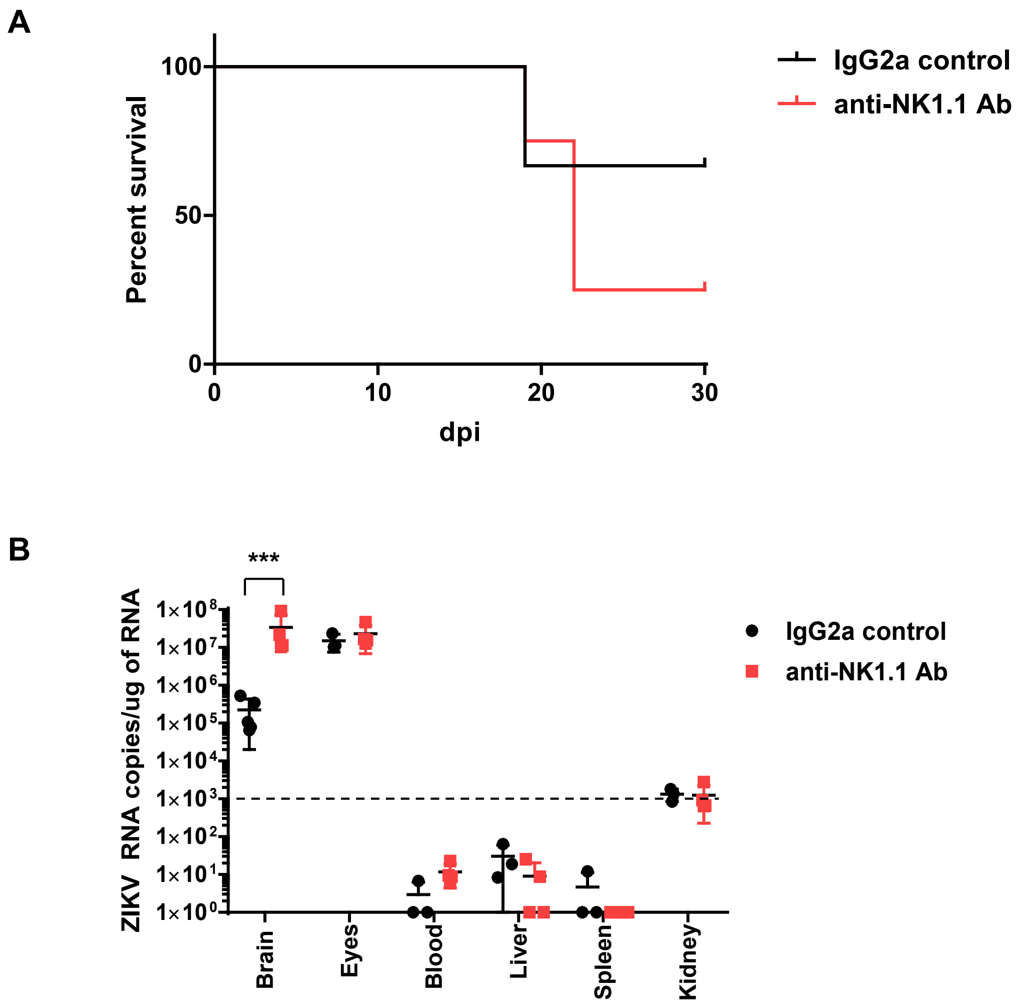

Supplemental Figure 8. NK cells are important for protecting mice from ZIKV-induced disease. (A and B) P1 WT mice were challenged with 1000 $TCID_{50}$/mL of ZIKV. From 3 dpi, 25 µg of IgG2a or anti-NK1.1 Abs were i.p. injected to ZIKV-infected WT mice every three days, and the survival was monitored for 30 days (n=3-4) **(A)**. Quantification of ZIKV RNA copies using real-time PCR was performed in the brain, eye, blood, liver, spleen, and kidney at 15 dpi (n=3-4) **(B)**.

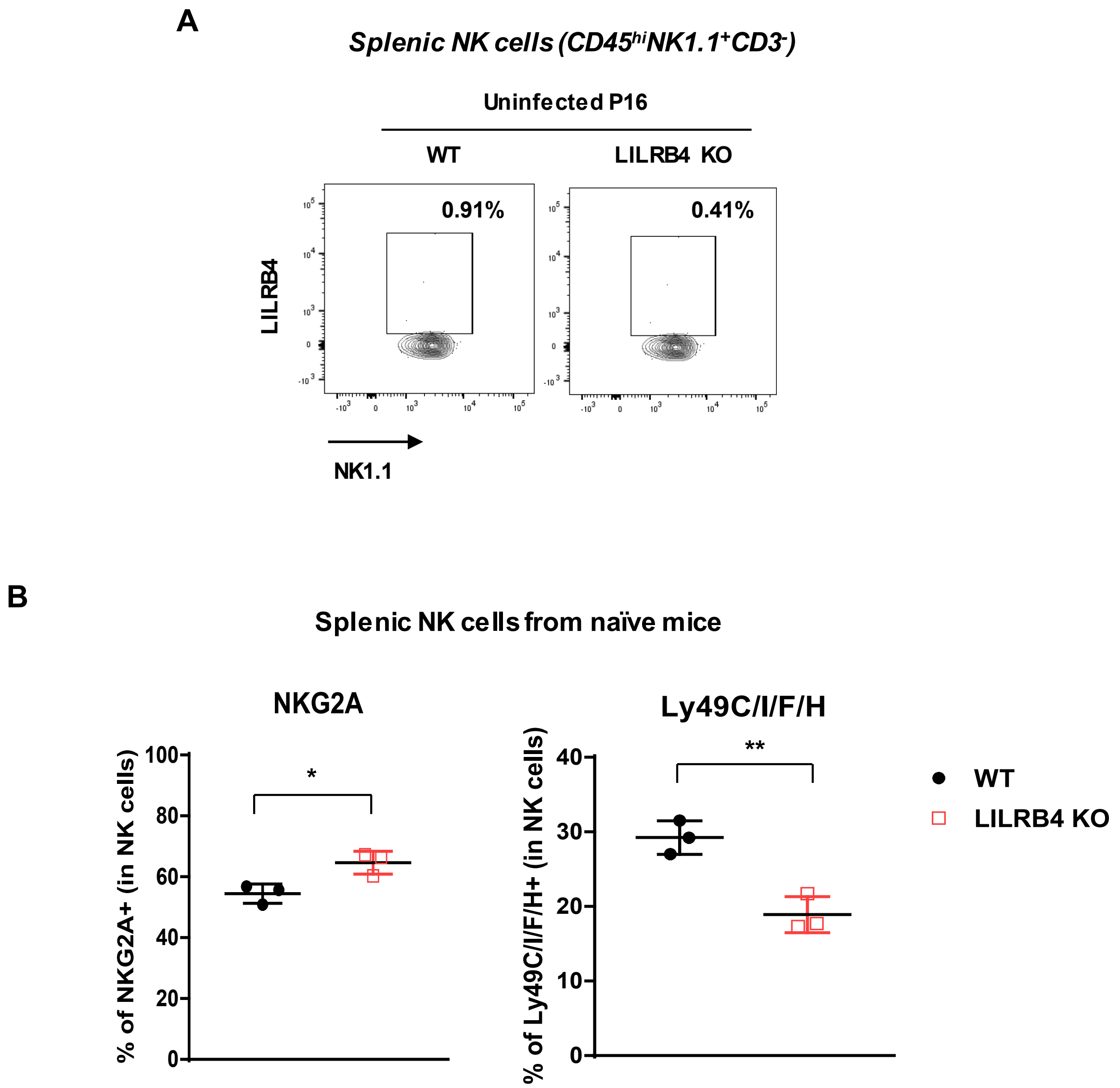

Supplemental Figure 9. Resting NK cells do not exhibit LILRB4 expression. (A and B) Splenocytes were isolated from naïve WT and LILRB4 KO mice at P16. Flow cytometry analysis determined the expression of LILRB4 **(A)**, NKG2A and Ly49C/I/F/H **(B)** on NK cells ($CD45^{hi}NK1.1^{+}CD3^{-}$). Data are representative of two independent experiments.

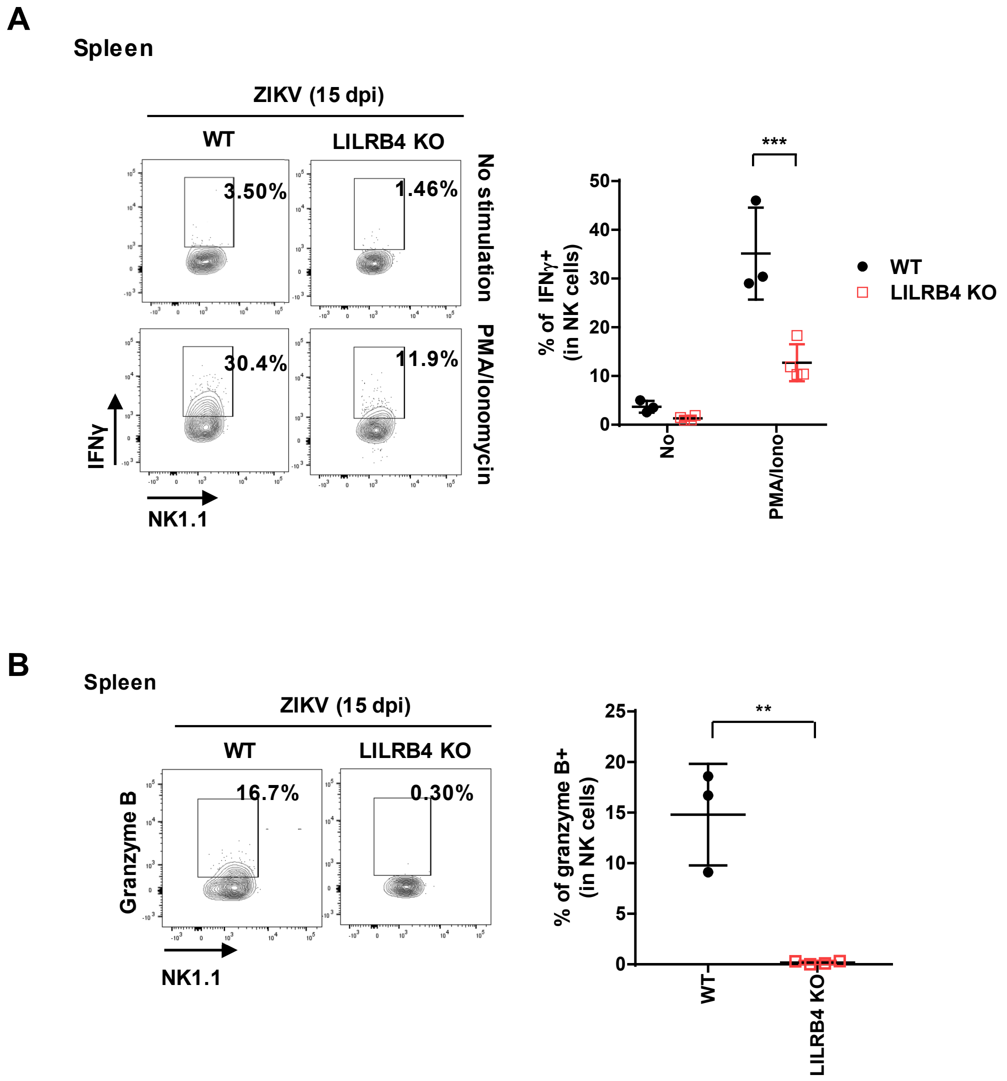

Supplemental Figure 10. LILRB4 deficiency impairs IFNγ and granzyme B production by splenic NK cells upon ZIKV challenge. (A and B) Cells were isolated from the spleen of ZIKV-infected WT and LILRB4 KO mice at 15 dpi, and then incubated with brefeldin A (10 μg/mL) with or without PMA (10 ng/mL) and ionomycin (1 μg/mL) for 3.5 h. The intracellular expression of IFNγ **(A)** and granzyme B **(B)** was determined by flow cytometry in NK cells ($CD45^{hi}NK1.1^{+}CD3^{-}$). The graphs show the percentages of NK cells expressing IFNγ **(A)** and granzyme B **(B)**.

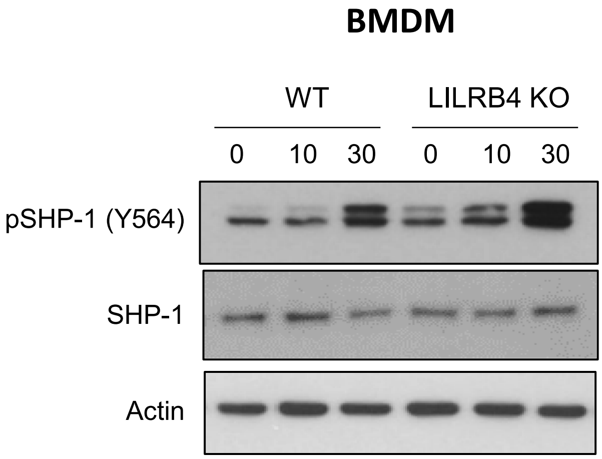

Supplemental Figure 11. LILRB4 deficiency upregulates SHP-1 phosphorylation in response to ZIKV. BMDM from WT or LILRB4 KO mice were incubated with ZIKV (MOI 1) for the indicated time. The levels of phosphorylated (Y564) and total SHP-1 were detected by Western blot analysis. Actin was used as an equal loading control for normalization. Data are representative of two independent experiments.

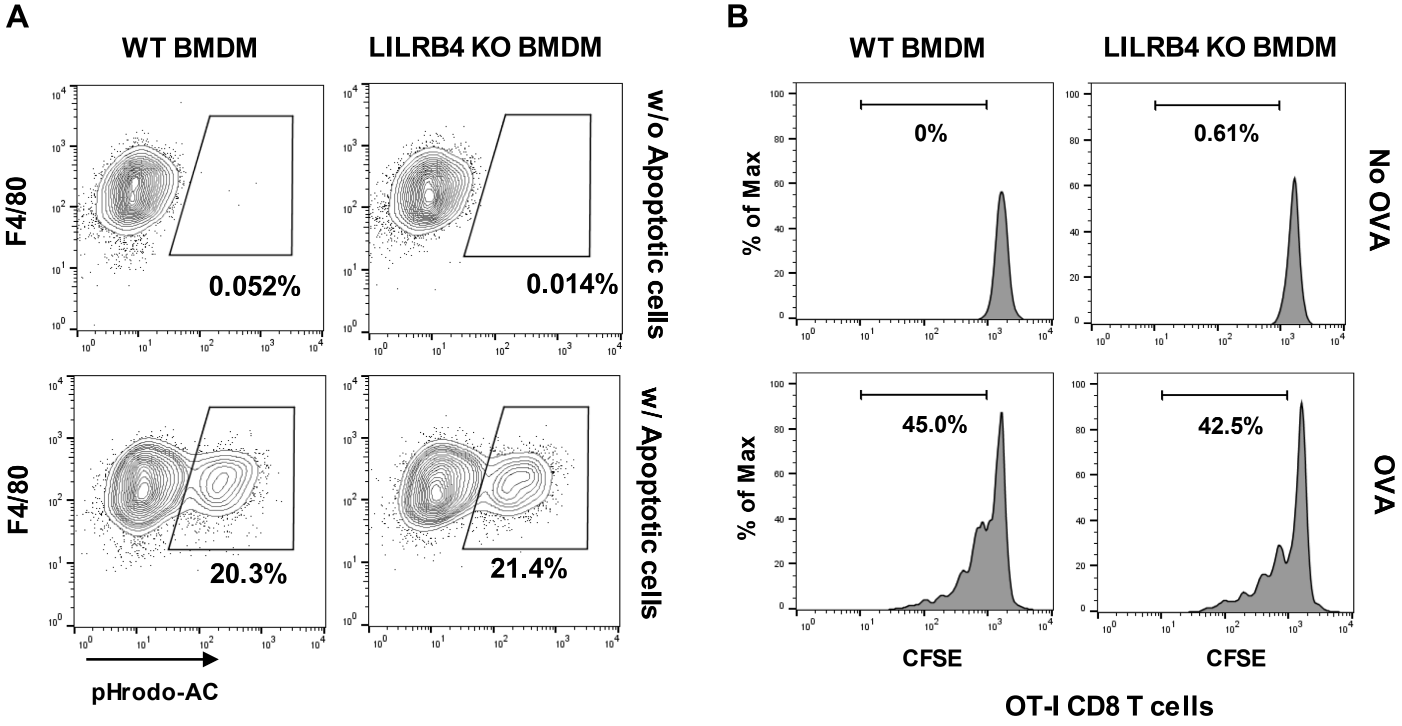

Supplemental Figure 12. LILRB4 deficiency does not affect phagocytic and antigen-presenting ability of macrophages. (A) BMDM from WT or LILRB4 KO mice were incubated with pHrodo-labeled apoptotic thymocytes at a 1:3 ratio and, after 60 min, the phagocytosis of apoptotic cells (AC) by F4/80$^+$ macrophages was determined by flow cytometry. The plots show representative results of the percentage of macrophages that engulfed AC (F4/80$^+$pHrodo$^+$ cells). **(B)** Proliferation of CFSE-labeled OT-I CD8+ T cells upon co-incubation with ovalbumin (OVA)-loaded apoptotic thymocytes and BMDM isolated from WT or LILRB4 KO mice for 4 days. The histograms illustrate representative results of CFSE dilution.

A

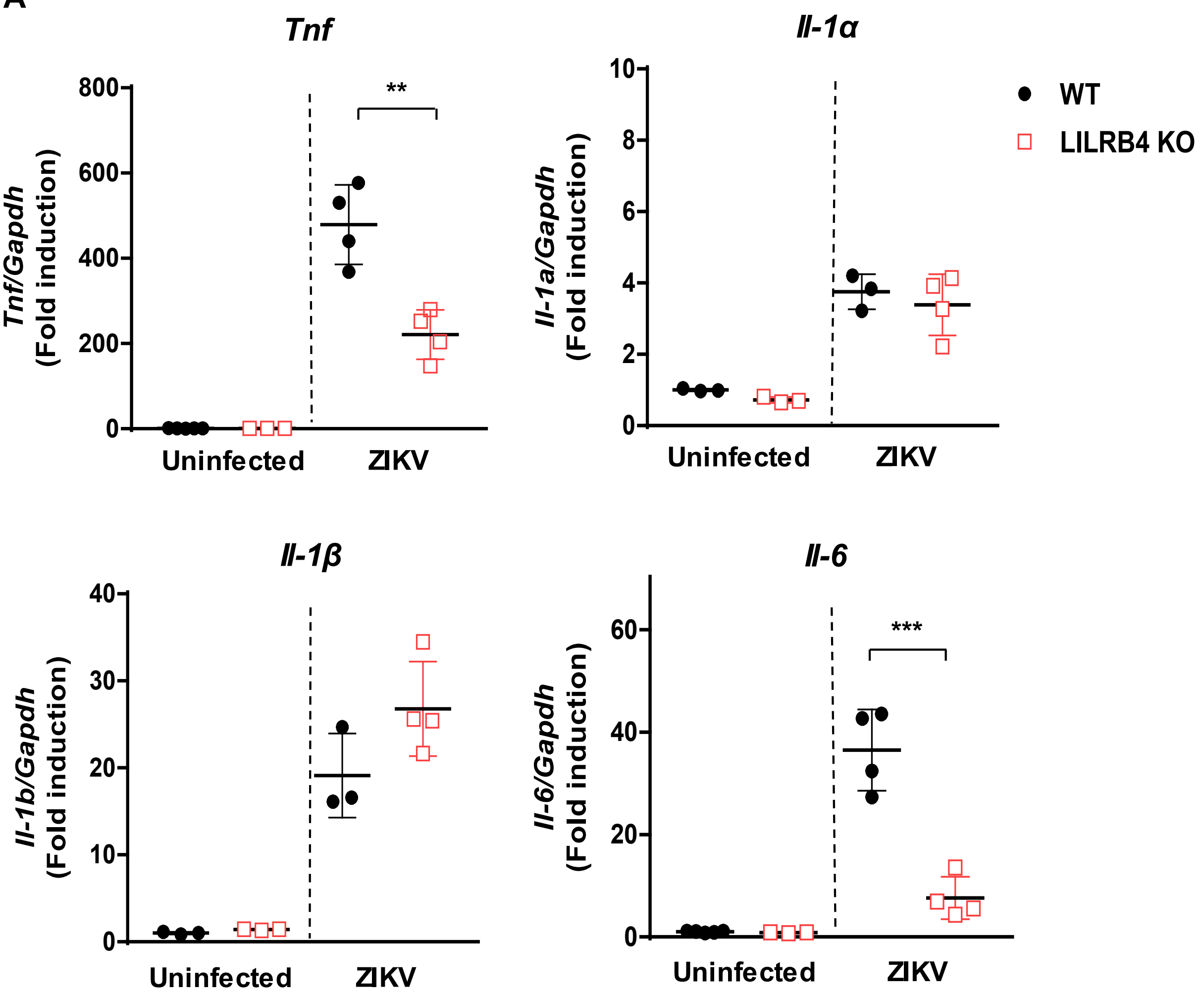

Tnf
Il-1α
Il-1β
Il-6
WT
LILRB4 KO
Tnf/Gapdh (Fold induction)
Il-1a/Gapdh (Fold induction)
Il-1b/Gapdh (Fold induction)
Il-6/Gapdh (Fold induction)
**
***
Uninfected
ZIKV

B

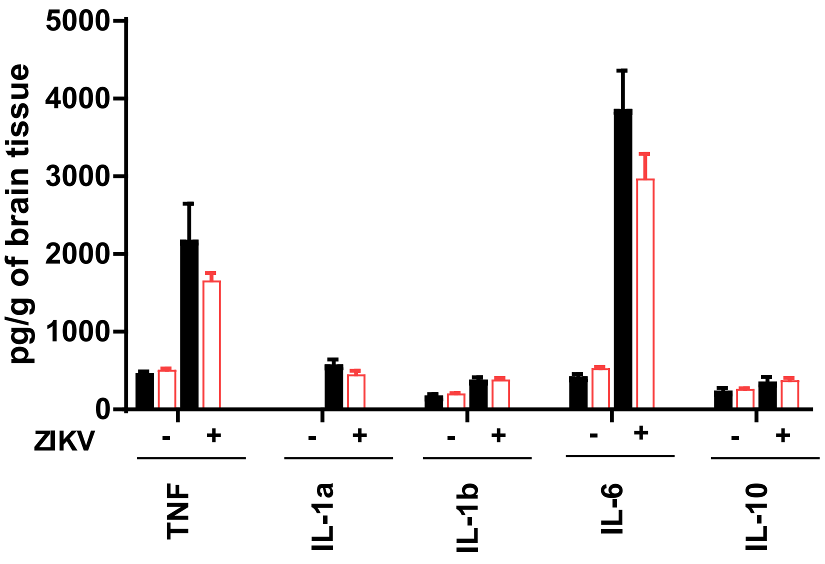

pg/g of brain tissue
5000
4000
3000
2000
1000
0
ZIKV
- +
TNF
IL-1a
IL-1b
IL-6
IL-10

Supplemental Figure 13. LILRB4 deficiency does not result in increased pro-inflammatory cytokine production. **(A)** *Tnf, Il-1α, Il-1β* and *Il-6* mRNA levels were determined in the brain of uninfected or ZIKV-infected WT and LILRB4 KO mice at 15 dpi using real-time RT PCR. **(B)** TNF, IL-1α, IL-1β, IL-6 and IL-10 protein levels were determined in the brain lysates from uninfected or ZIKV-infected WT and LILRB4 KO mice at 15 dpi using luminex. Data shown as means ± S.D. (n=2-4). $^{**}P < 0.01$, $^{***}P < 0.001$

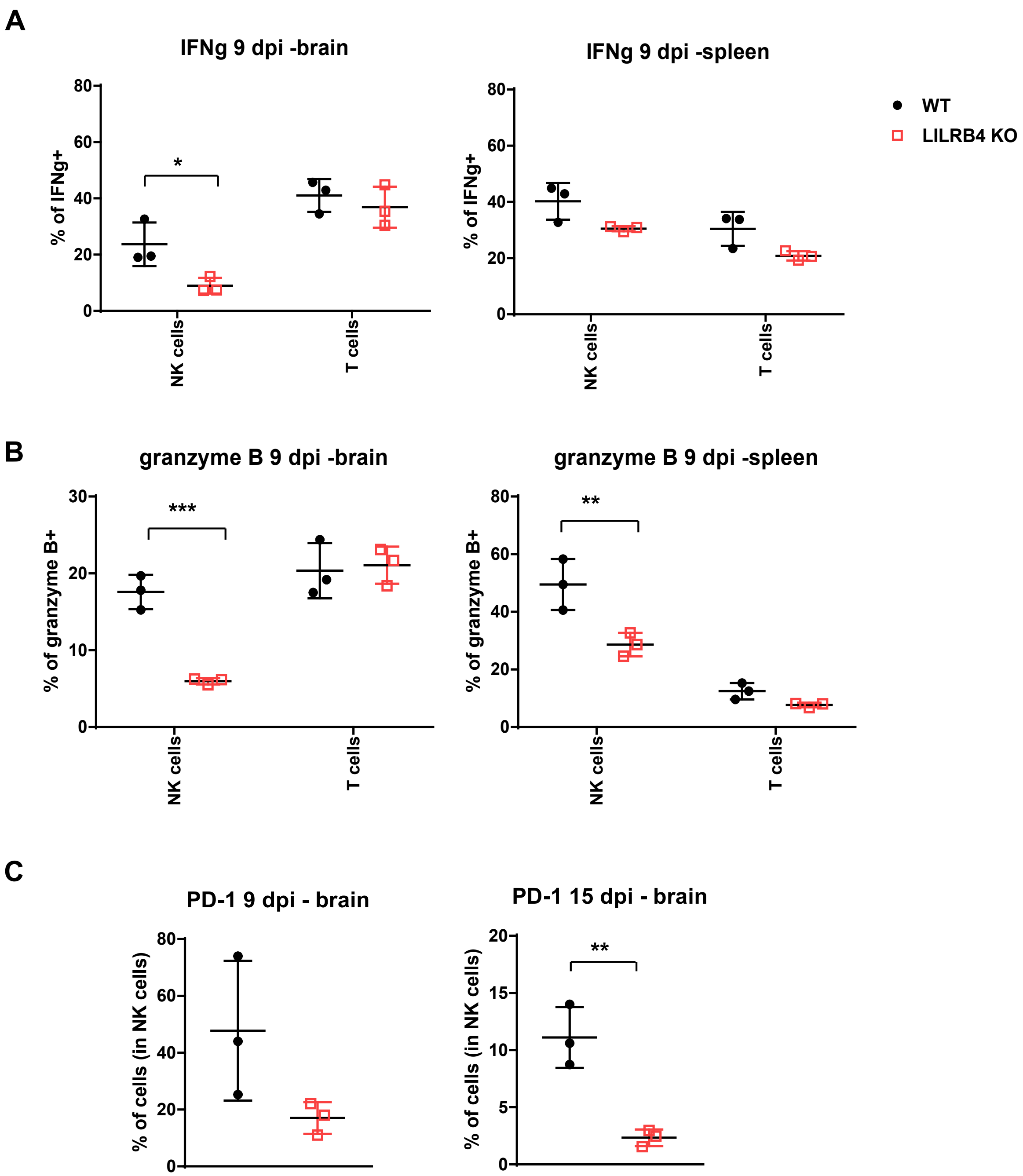

A
IFNg 9 dpi -brain
% of IFNg+
0
20
40
60
80
*
NK cells
T cells
IFNg 9 dpi -spleen
% of IFNg+
0
20
40
60
80
NK cells
T cells
WT
LILRB4 KO
B
granzyme B 9 dpi -brain
% of granzyme B+
0
10
20
30
***
NK cells
T cells
granzyme B 9 dpi -spleen
% of granzyme B+
0
20
40
60
80
**
NK cells
T cells
C
PD-1 9 dpi - brain
% of cells (in NK cells)
0
20
40
60
80
PD-1 15 dpi - brain
% of cells (in NK cells)
0
5
10
15
20
**

Supplemental Figure 14. Less IFNγ and granzyme B expression by NK cells is not due to exhaustion. (A) Cells were isolated from the brain and spleen of ZIKV-infected WT and LILRB4 KO mice at 9 dpi, and then incubated with brefeldin A (10 μg/mL) with or without PMA (10 ng/mL) and ionomycin (1 μg/mL) for 3.5 h. The intracellular expression of IFNγ was determined by flow cytometry in NK cells ($CD45^{hi}NK1.1^{+}CD3^{-}$) and T cells ($CD45^{hi}CD3^{+}NK1.1^{-}$). The graphs show the percentages of NK and T cells expressing IFNγ. **(B)** Cells were isolated from the brain and spleen of ZIKV-infected WT and LILRB4 KO mice at 9 dpi, and then incubated with brefeldin A (10 μg/mL) for 3.5 h. The intracellular expression of granzyme B was determined by flow cytometry in NK cells ($CD45^{hi}NK1.1^{+}CD3^{-}$) and T cells ($CD45^{hi}CD3^{+}NK1.1^{-}$). The graphs show the percentages of NK and T cells expressing granzyme B. **(C)** Cells were isolated from the brain of ZIKV-infected WT and LILRB4 KO mice at 9 and 15 dpi, and the expression of PD-1 was determined by flow cytometry in NK cells ($CD45^{hi}NK1.1^{+}CD3^{-}$). The graphs show the percentages of NK cells expressing PD-1. Data are representative of three independent experiments (n =3, each). $*P < 0.05$, $**P < 0.01$, $***P < 0.001$
